# Soundscape Assessment of Aircraft Height and Size

**DOI:** 10.3389/fpsyg.2018.02492

**Published:** 2018-12-18

**Authors:** Gianluca Memoli, Giles Hamilton-Fletcher, Steve Mitchell

**Affiliations:** ^1^School of Engineering and Informatics, University of Sussex, Brighton, United Kingdom; ^2^Environmental Resources Management, London, United Kingdom

**Keywords:** soundscapes, aircraft, height perception, size perception, multisensory perception, questionnaire design, survey development, interviews

## Abstract

It is accepted knowledge that, for a given equivalent sound pressure level, sounds produced by planes are worse received from local communities than other sources related to transportation. Very little is known on the reasons for this special status, including any interactions that non-acoustical factors may have in listener assessments. Here we focus on one of such factors, the multisensory aspect of aircraft events. We propose a method to assess the visual impact of perceived aircraft height and size, beyond the objective increase in sound pressure level for a plane flying lower than another. We utilize a soundscape approach, based on acoustical indicators (dBs, *L*_A, max_, background sound pressure level) and social surveys: a combination of postal questionnaires (related to long-term exposure) and field interviews (related to the contextual perception), complementing well-established questions with others designed to capture new multisensory relationships. For the first time, we report how the perceived visual height of airplanes can be established using a combination of visual size, airplane size, reading distance, and airplane distance. Visual and acoustic assessments are complemented and contextualized by additional questions probing the subjective, objective, and descriptive assessments made by observers as well as how changes in airplane height over time may have influenced these perceptions. The flexibility of the proposed method allows a comparison of how participant reporting can vary across live viewing and memory recall conditions, allowing an examination of listeners' acoustic memory and expectations. The compresence of different assessment methods allows a comparison between the “objective” and the “perceptual” sphere and helps underscore the multisensory nature of observers' perceptual and emotive evaluations. In this study, we discuss pro and cons of our method, as assessed during a community survey conducted in the summer 2017 around Gatwick airport, and compare the different assessments of the community perception.

## Introduction

It is well-accepted that, for a given sound pressure level (SPL), aircraft are perceived by local communities to be more annoying than other transportation sources (WHO, 2009). This special status of aircraft-generated sounds has been evolving with time, so that recent studies identified an ongoing increase in sensitivity to aircraft sounds in communities: for the same sound-pressure level, these studies record a larger percentage of annoyed respondents than, say, 10 years ago (Guski et al., 2017). The reasons for this increase are still unclear: part of the research community attributes this to the “rate of change” in the number of aircraft movements (MVA-Consultancy, 2007) and in the composition of aircraft fleets (Janssen et al., 2011; Guski, 2017), while others report a general change in the attitude toward planes and an increase in the weighting of non-acoustical factors (Bartels et al., 2015).

Recent estimates attribute 66–75% of the variation in recorded perception to non-acoustical factors (Guski, 1999; Arras et al., 2003; Nillson et al., 2007). However, while factors like demographics, occupation, self-reported sensitivity, feeling of being in control are broadly covered in the literature, aspects such as visual perceptions, expectations, and judgments regarding these sound sources are rarely covered.

In this context, different airports, in the United Kingdom (Redeborn and Lake, 2016) and elsewhere (Schreckenberg et al., 2016; Hiroe et al., 2017), have recorded in their local communities evidence of a specific non-acoustical factor, usually worded as “planes are flying lower than before.” As reported in Gatwick's Independent Arrivals Review (Redeborn and Lake, 2016), this perception often finds no correspondence in objective data, which show only negligible changes in the height distribution of arriving aircraft, in their average arriving paths or in the measured sound pressure levels.

To the soundscape scientist, this apparent discrepancy between objective and subjective heights suggests a combined effect of visual and acoustic factors in the perception of residents under arrivals routes. Similar cross-modal interaction on acoustic judgements has been highlighted in the context of quiet areas (Pheasant et al., 2007) but, to the authors' best knowledge, has not been properly investigated for aircraft sounds so far. This study is a first attempt to address this aspect of community perception.

Here we propose a method, based on the combination of measurements and social surveys, to address questions like “is aircraft height perceived by individuals reasonably accurately?” and “is there a correlation between aircraft size and height perception?” In a context where it is not clear what causes the reported effect on perception, we propose to run simultaneously one measurement campaign and two social surveys: the first, based on postal questionnaires, 30–40 min long and oriented to long term perception, and the second, based on 15-min face-to-face interviews and focused on assessing perception contextually to the planes passing during the interview. We discuss the design of the two social surveys and their interplay, highlighting how they offer two different but complementary windows on the perception of local communities.

Finally, we discuss pro and cons of the method following a preliminary test on about 200 residents around Gatwick in the summer of 2017.

## Materials and Methods

### From Research Hypotheses to Survey Design

According to Frankfort-Nachmias et al. (2015), the design of a social survey requires at least one question (i.e., “is there a non-acoustic impact of aircraft height and size on acoustic perception?”) and one hypothesis. At the start of this work, we had two.

The first hypothesis, suggested in Gatwick's Independent Arrivals review (Redeborn and Lake, 2016), attributes the perceived effect to the changing fleet makeup, with larger, but similar proportioned planes being increasingly used over time: an argument used by other studies to explain an increased awareness toward plane-originated sounds (Guski, 2017). This suggests that observers may believe the planes to be closer due to their larger visual size during observation and, potentially, due to a potential contribution on the acoustic side (i.e., larger aircraft may appear even bigger due to increased SPL). This hypothesis is mainly visual and can be assessed by a survey containing appropriate questions on height and size only and by a thorough analysis of aircraft movements and physical dimensions (e.g., from radar tracks).

The second hypothesis, proposed by the authors, was inspired by a well-known report into soundscape research (Payne et al., 2009), which highlighted the multisensory character of what are normally labeled simply as “auditory” experiences. The “soundscape approach” suggests evaluating the interaction between the sounds, the visual size, and the spatial height of passing planes.

If such a multisensory interaction between vision, perception, and interpretation of aircraft sounds exists, this should not be balanced: there is in fact a stronger tendency to favor visual information on acoustic stimuli, rather than the reverse (Posner et al., 1976; Bregman, 1990). In this context, the intrinsic difficulty of judging the height of a passing plane would generate an ambiguity, which is resolved by an increased reliance on alternate senses. For testing this second hypothesis, height-specific questions needed to be accompanied by sound perception ones, like those in the standardized surveys (Fields et al., 2001).

Aircraft sounds, however, can be experienced both indoors and outdoors. Height effects on perception can come from long-term memory (e.g., an opinion built on the repeated passage of lower aircrafts) or short-term judgements (e.g., the occasional passage of an outlier aircraft, sedimented in the memory). To remove these ambiguities, in this study we use in parallel two different interaction modalities: a 40 min long questionnaire, focused on long-term perceptions, and a 15-min questionnaire, targeting short-term judgements. Inspired by the high response rate (60%) recently achieved near Narita (Hiroe et al., 2017), we decided to deliver the 40-min questionnaires by post and the 15-min one during semi-structured interviews. The postal questionnaire was designed to be completed by the participants unassisted and indoors. The semi-structured interviews, designed to be run with a researcher, were targeted to participants outdoors and included a component of “plane spotting,” which was used to assess perceptual judgements “there and then.”

We designed the two surveys to be interconnected, so that some key questions were repeated, in view of a future comparison. As an example, while exposure outdoor was primarily assessed by interviews, the postal survey also contained two key questions related to aircraft perception outdoors. When possible, we maintained the ICBEN 11-point numeric scale in the postal questionnaires and the 5-point ICBEN verbal scale in the interviews (Fields et al., 2001). A similar choice was taken near Narita (Hiroe et al., 2017) and the two scales were compared using recent guidelines (Brink et al., 2016).

Finally, the two social surveys were designed to be assisted by a measurement campaign, also to be ran in parallel, with the goal of assessing the acoustic climate in the selected survey areas, but also of associating acoustic indicators like *L*_A, max_ and *SEL* (WHO, 2009) to the planes observed during the field interviews. Measurements of plane trajectories (to assess visual distances^1^ and real heights) could be done in post-processing, linking the exact time of the passage with the data from flight-tracking apps like *FlightRadar24* or *CASPER*.

### Characterization of the Survey Areas

**Figure 1:**
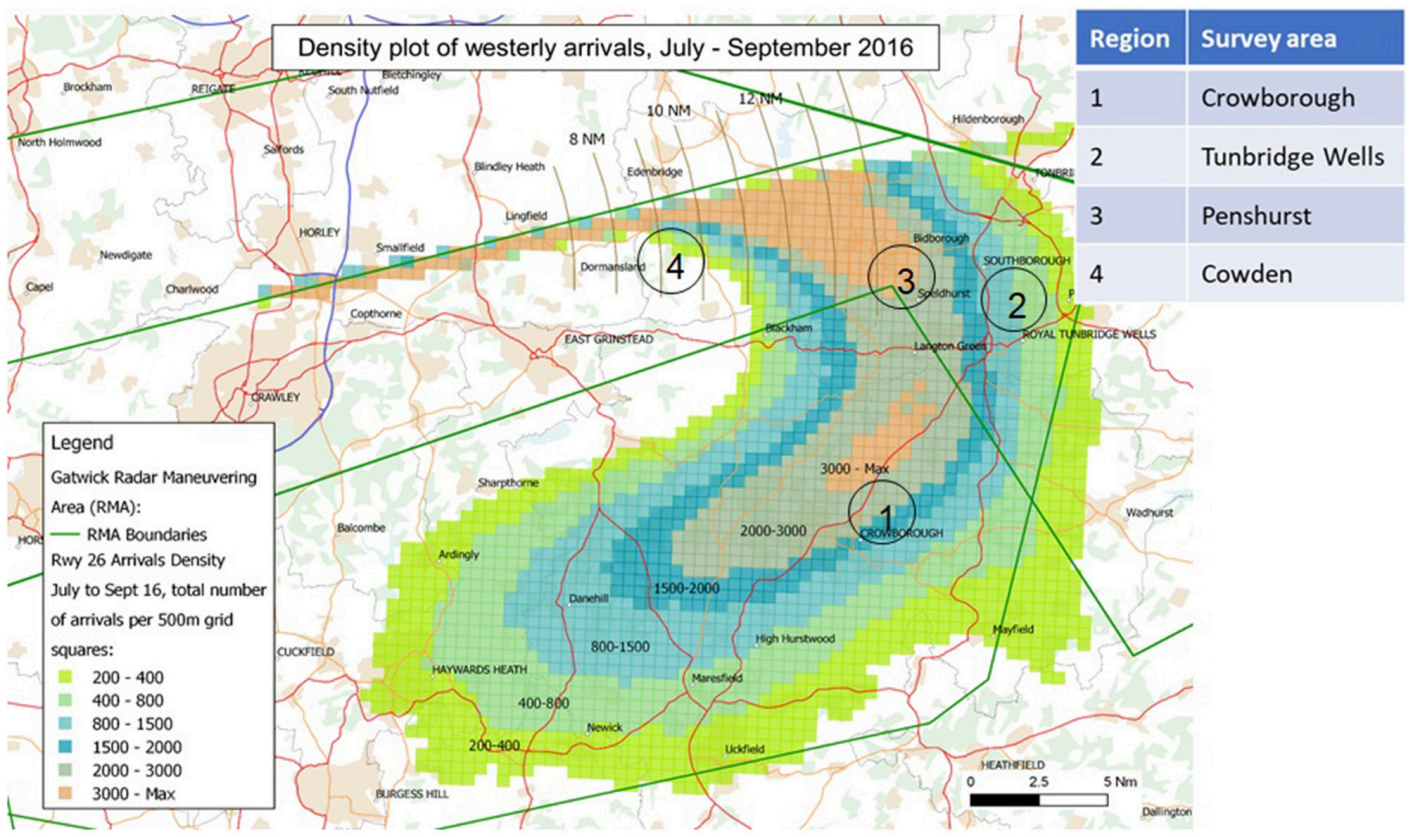
Geographical position of the survey areas for this study, relative to the arrivals distribution to the east of Gatwick in 2016. Contours (courtesy of Gatwick Ltd.) refer to the overall number of planes tracked over a specific location in summer 2016. Areas are not in scale.

We tested our method in the summer of 2017, when the number of flights reaches its peak. In the period 28/8–30/9, we focused on three locations to the east of Gatwick airport, along the main arrival path (“westerly arrivals,” see Figure 1): Crowborough, Penshurst and the center of Tunbridge Wells. Each of these three areas was characterized by a different average aircraft altitude over the ground level (as measured by Gatwick using radar tracks) and contained about 300 households. Figure 1 also shows the site of Cowden, which was used as a control, with 200 households.

For the purposes of this study, we will assume that the height distribution of the planes passing over each survey areas is very close to a Gaussian^2^. This hypothesis defines the first statistical parameter with which to characterize each area i.e., the mean height, which corresponds to the height of the most frequently observed plane. As second descriptive parameter, instead of using the standard deviation, we used the height of the lowest plane (defined as the 1st percentile of the height distribution). Having received from Gatwick the numerical height distributions relative to summer 2016 for the different locations (Helios, 2016), we therefore characterized each of the survey areas with two parameters: the height of the “most frequent” plane and that of the “lowest” plane (see Table 1).

**Table 1:** Parameters characterizing the 4 locations surveyed in the Summer of 2017. Heights are referenced to the local ground level. Source: (Helios, 2016).

**Location**	**Lowest plane/ft**.	**Most frequent plane/ft**.
Crowborough	2390	5257
Tunbridge Wells	2590	4683
Penshurst	2019	4236
Cowden	1748	3818

In terms of population, while Cowden and Penshurst are small villages surrounded by countryside, Crowborough and Tunbridge Well are more urbanized areas. Simply walking through the areas shows that most of the residents live in detached or semi-detached houses. According to the most recent census (Office for National Statistics, 2011), the overall population living in the selected villages and towns could be stratified as follows:

- Age according to census: 18–24 (8%), 25–34 (18%), 35–44 (18%), 45–54 (19%), 55–64 (15%), 65, and over (23%).
- Gender according to census: males (51%), females (49%).

In terms of exposure to aircraft sounds, the selected areas are at least 18 km away from the local airport: a distance much larger than the ones typically surveyed in other studies (MVA-Consultancy, 2007; Civil Aviation Authority, 2017) and beyond the lowest contour (57 dBA *L*_*Aeq*, 16*h*_ by day) of the local noise map (Environmental Research Consultancy Department, 2017). It was therefore necessary to assess acoustical indicators by direct measurements.

Gatwick airport contributed to this study by deploying a mobile acoustic monitor in each of the 4 survey areas. The monitors (Larson Davis, type 870) were mounted inside a weatherproof metal cabinet and connected to an outdoor microphone located at about 4.0 m from the ground (ISO 1996-2, 2017). The monitors were programmed to record all noise events, but those with *L*_*Aeq*_≥55dBA (and lasting at least 10 s) were correlated automatically with details of the aircraft and its flight path using a Noise Track Keeping (NTK) system. Values of *L*_A, max_ were acquired using a Slow (1 s) time constant.

In addition, a calibrated class I spectrum analyzer (Norsonic 121) was present during most of our field interviews, with its 1/2″ microphone mounted on a tripod at 1.5 m from the ground (ISO 1996-2, 2017). These measurements were aimed at planes with *L*_A, max_ < 55 dBA, for which (we thought) the visual component (i.e., the aircraft height, size, and visual distance) could distinguish planes characterized by the same acoustics. Here, the assignment of *L*_A, max_ to a specific airplane was performed in post-processing, by synchronizing the measurement with the radar tracks as reported by CASPER (Casper, 2017).

We did not apply any correction for ground reflections (ISO 1996-2, 2017) to the Norsonic measurements, because most of the time the tripod with the microphone was on soft ground (grass), all the interviews were taken in the same (favorable) weather conditions, our acoustic sources were very far from the microphone, and we only used the *L*_A, max_ of events as they happened.

Our measurements showed that, in each of the areas, plane sounds contributed with an estimated^3^ value of *L*_*DEN*_ between 47 and 50 dBA, while background sounds (i.e., as given by the level that was overcome 90% of the time, or *L*_90_) were between 35 and 37 dBA. In summary, all the survey areas were subject to the same exposure to aircraft sounds, in terms of average energy levels.

## The Postal Survey

### Recruitment

A package was sent to randomly selected residents in each survey area (50% of the households), including a pre-paid return envelope and three items (an introductory letter, a consent form and the postal questionnaire), anonymized with a unique ID, in the format “Y-XXXX” where “Y” identified the survey area and “XXXX” is a random number.

The consent form was based on a template produced by the Sciences & Technology Cross-Schools Research Ethics Committee at Sussex and explained how returning the questionnaire was considered an “explicit” act of consent to take part in the study (European Commission, 2011) and to treat the answers anonymously, unless further consent was given (e.g., volunteering for a follow-up, see below). It also detailed how data would be stored and reported instructions on how to withdraw participation.

As a novelty compared to previous studies, we provided an additional mechanism, at the end of the postal questionnaire, aimed at recruiting a small control set of participants. Postal responders could volunteer also to be interviewed (by appointment), in their garden or in a park nearby, thus providing an immediate check between the two interaction modalities (i.e., the postal and the face-to-face interviews).

### Questionnaire Design

The postal questionnaire consisted in 80 questions: a combination of the well-established, key questions from technical specification ISO/TS 15666:2003 (Fields et al., 2001; ISO/TS 15666:2003, 2017) and of a set of custom questions, specific to assessing long-term perception of aircraft height/size (see below). The postal questionnaire used in this study can be found attached as Annex 1 and a detailed description of its sections has been added to the Supplementary Material S1.

Whether by postal questionnaires, filled at home (Janssen et al., 2011; Hiroe et al., 2017), interviews by telephone (Schreckenberg et al., 2016) or in-person appointments (MVA-Consultancy, 2007; Civil Aviation Authority, 2017), the surveys based on ISO/TS 15666:2003 measure the impact of unwanted sounds on perception in terms of the single parameter “annoyance,” evaluated over long periods and at home (ISO/TS 15666:2003, 2017). They share a variant of the same question (“Thinking about the last 12 months, when you are at home, how much does noise from [planes, traffic, rail] bother, disturb, or annoy you?”) and their results are quantitatively assessed using either a 5-point verbal scale (“not-at-all” to “extremely”), for use with verbal questions, or an 11-point numerical scale (0–10), for use in written questions (Fields et al., 2001).

There is additional difficulty in adding height-specific questions to such a survey, as the exact nature of forming expectations around height may be informed via visual inspection or auditory influences, and the mere fact of asking participants to evaluate the acoustic environment may alter their attention and listening strategy^4^ (Truax, 2001). Unwanted effects were mitigated by allowing neutral or positive responses even for what are usually defined “unwanted sounds” (i.e., “noise”) in standard questions (Fields et al., 2001). When possible, we also maintained the same wording and positional sequence of questions (Abe et al., 2006). We decided, however, to stick to the traditional single dimension of “annoyance” (which has a negative connotation in itself), even if more recent studies demonstrate that a multi-dimensional analysis may be more appropriate (Schreckenberg et al., 2017).

#### Height Scale

**Figure 2:**
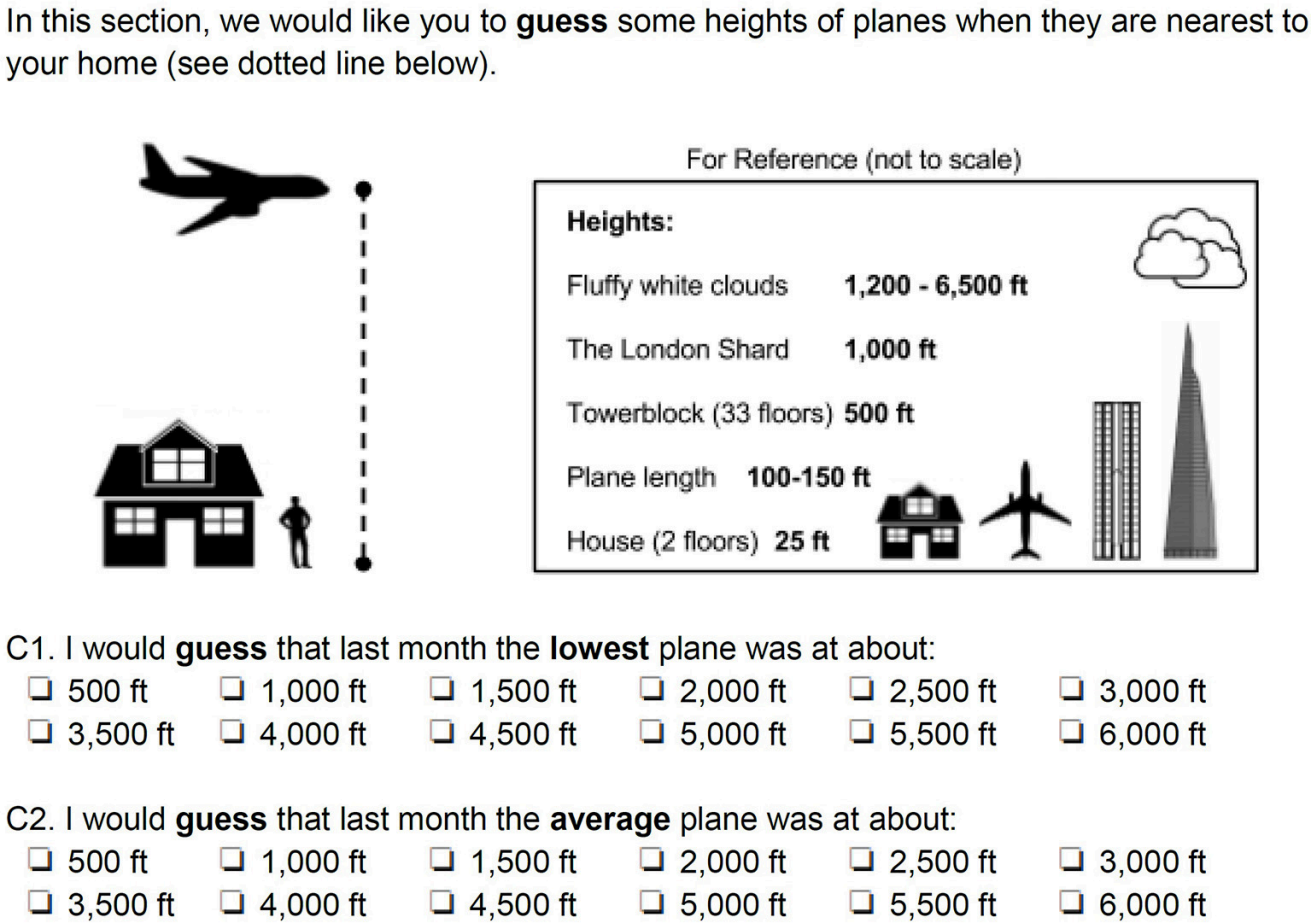
Quantitative assessment of the perceived height of planes in the postal questionnaire.

**Figure 3:**
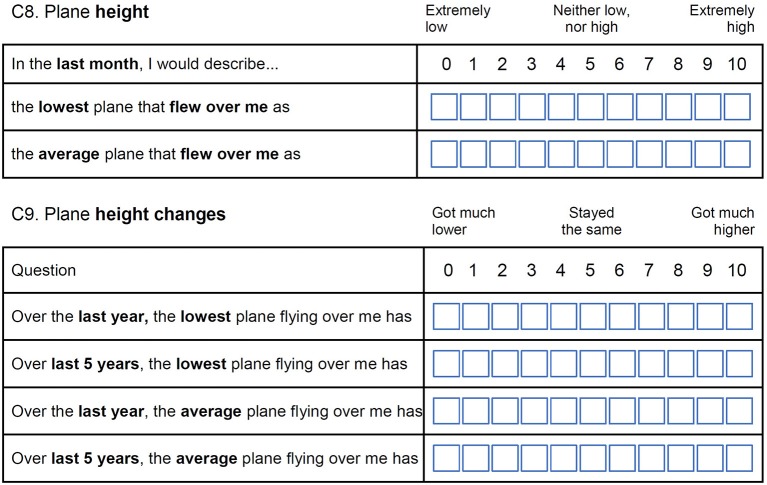
Qualitative assessment of the perceived height of planes in the postal questionnaire.

Since the postal questionnaire refers to the memory of the respondent, it is not possible to compare directly a perceptive judgement with the real height of a passing plane: the comparison can only be done with statistical quantities. As shown in Figure 2, we decided to introduce two perceived quantities–i.e., the “average plane” and the “lowest plane”–without further instructions for the respondents. Nevertheless, as discussed in section Result and Discussion, this apparently free choice linked very clearly to a specific perception of the participants. In the postal questionnaire, we assess height in two ways:

- Quantitatively, asking the respondent a numerical judgement on the height of the “average” plane and the “lowest” plane flying over his/her home (questions C1 and C2 in Figure 2).
- Qualitatively, asking the participant a perceptual judgement on the average/lowest plane flying over his/her home (question C8). We also ask whether the height of the lowest/average plane had changed compared to 1 year or 5 years ago (question C9 in Figure 3).

As shown in Figure 2, during the initial testing phase for the postal questionnaire, we realized that height assessment required some visual reference, either in the memory of the observer (e.g., famous local landmarks like the Shard or a tower block) or, better, something that could be found on the scene. We initially thought of the clouds but discarded the idea once we saw that their potential height range (1,200–6,500 ft.) is weather-dependent. We then realized that the only object always on the scene is the plane itself, so we added one to the graphical scale. Equally important in Figure 2 is the presence of a dotted vertical line, to resolve any potential ambiguity between “visual distance” (i.e., the distance between the observer and the passing plane, which may be at an angle) and “height” (which may not be close to the observer).

#### Size Scale

**Figure 4:**
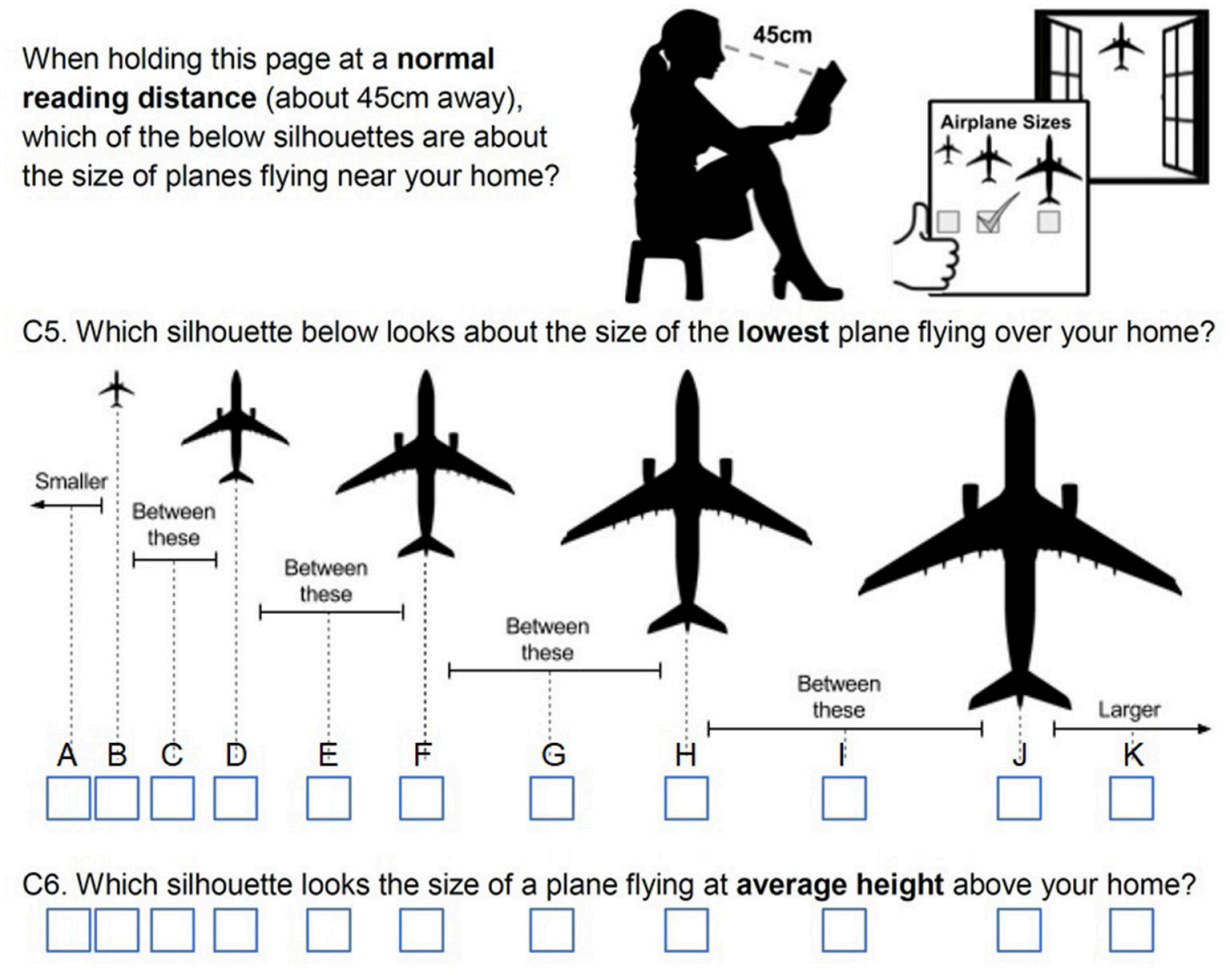
Qualitative assessment of the perceived size of planes in the postal questionnaire. This chart allows for a quantitative assessment when the distance between the eye and the chart is known.

Figure 4 shows the graphical scale that accompanies questions on size (C5 and C6) in the postal questionnaire, with the instructions to use it and the wording of the relative questions.

For assessing size, we wanted a method that could be used with as little guidance as possible and that could be valid for different visual distances. Eventually, we took inspiration from astronomy, where the size of a far star is assessed measuring its image on the eyepiece of the telescope, and devised a method based on the visual angle i.e., the amount of space that an image will subtend on the retina (Swearer, 2011). For a fixed object size, the visual angle depends on the distance between the object and the observer (i.e., the visual distance), so that larger distances lead to smaller visual angles. Similarly, for a fixed visual distance, larger objects lead to larger visual angles.

This method, which appears qualitative, becomes quantitative when the distance between the eye and the reference is known. We therefore put at normal reading distance (45 cm) the silhouettes of an A330^5^, scaled at sizes between 0.1 and 5 cm (see Figure 4) and asked the participant to select the one that appeared closest to either the average or the lowest plane. This assessment, together with the visual distance between the observer and the passing plane (that can be evaluated from flight tracks), gives a “perceived plane size,” which can then be compared with the true size (from flight tracks).

Table 2 shows a practical reference for size assessment, based on the last plane in Figure 4 being 5 cm long. As an example of using Table 2, an A320 flying at 5,200 ft. just above the observer (visual distance is 5,200 ft.) should be seen as “size C” (row: A320, column: the closest class to 5,200 ft.), while should be perceived as “size D” when flying at 3,800 ft.

**Table 2:** Visual distance at which different planes enter a new size class (in 1000s of feet).

**Visual Plane Size (45 cm away)**											
	0.1 cm	0.5 cm	1 cm	1.5 cm	2 cm	2.5 cm	3 cm	3.5 cm	4 cm	4.5 cm	5 cm
Class	A	B	C	D	E	F	G	H	I	J	K
A319	50.0	10.0	5.0	3.3	2.5	2.0	1.7	1.4	1.2	1.1	1.0
A320	55.5	11.1	5.5	3.7	2.8	2.2	1.8	1.6	1.4	1.2	1.1
737–800	58.3	11.7	5.8	3.9	2.9	2.3	1.9	1.7	1.5	1.3	1.2
A321	65.7	13.1	6.6	4.4	3.3	2.6	2.2	1.9	1.6	1.5	1.3
A330	94.0	18.8	9.4	6.3	4.7	3.8	3.1	2.7	2.3	2.1	1.9
777–200	94.0	18.8	9.4	6.3	4.7	3.8	3.1	2.7	2.4	2.1	1.9
787	92.7	18.5	9.3	6.2	4.6	3.7	3.1	2.6	2.3	2.1	1.9

*According to this chart, an A320 flying 2,200 feet above the observer is seen as class F i.e., the same size as an A330 flying at a visual distance of 3,700 ft. Visual distance at which the plane is seen under the same angle as at 45 cm*.

The uncertainty related to this method depends mainly on the distance between the reference chart and the eye of the observer. Short-sighted participants, for instance, would tend to keep the reference chart further away. Equally, as confirmed later observing participants during the interviews, long-sighted participants tend to keep it closer. During the testing phase of the postal questionnaire, we estimated an uncertainty of ±5 cm, which introduces an uncertainty of approximately one step in the perception scale (i.e., a correct judge of size, holding the visual chart at 40 cm instead than 45 would judge the planes to be one size larger).

The difficulty in making independent size and height judgements is demonstrated by the effect known as “the moon illusion.” It is in fact undisputed that the moon over the horizon appears to be larger than the moon high in the sky (Hershenson, 1989a). This difference in the perception of the size of the moon is illusory: while the perceived size is different at different elevations above the horizon, the physical stimulus that is produced by the light reflected from the moon (i.e., the visual angle at the eye of the viewer) does not change. If a similar effect applies to planes, the perceived size should get larger as the plane gets closer to the horizon (i.e., as the angle to the observer increases).

### Results and Discussion

For this study, we will only report the results concerning the perception of height and size and their relationship with noise measurements and annoyance. Further details can be found in a public report on the Gatwick website (Memoli et al., 2018).

#### Demographics

In the selected areas, we collected 112 postal questionnaires (20% response rate). The sample was stratified as follows:

- Self-declared age (postal): 18–24 (1%), 25–34 (3%), 35–44 (12%), 45–54 (15%), 55–64 (31%), 65, and over (38%).
- Self-declared occupation: full-time employed (27%), part-time employed (9%), retired (43%), home/carer (8%), other (12%), prefer not to say (2%).
- Self-declared type of home: detached house/bungalow (55%), semi-detached house/bungalow (32%), other (13%).

According to the age distribution, even if the sample was small, it was representative of the demographics in the area–as assessed by Office for National Statistics (2011). A good part of the postal respondents was over 55, while the younger side of the age distribution (i.e., 18–24) was much less represented. This was either due to the request, at the start of the postal questionnaire, of selecting “the person who spends most time at home” as representative of the household or to a concentration of aged residents in the specific survey areas.

#### Perception of Height and Size

**Figure 5:**
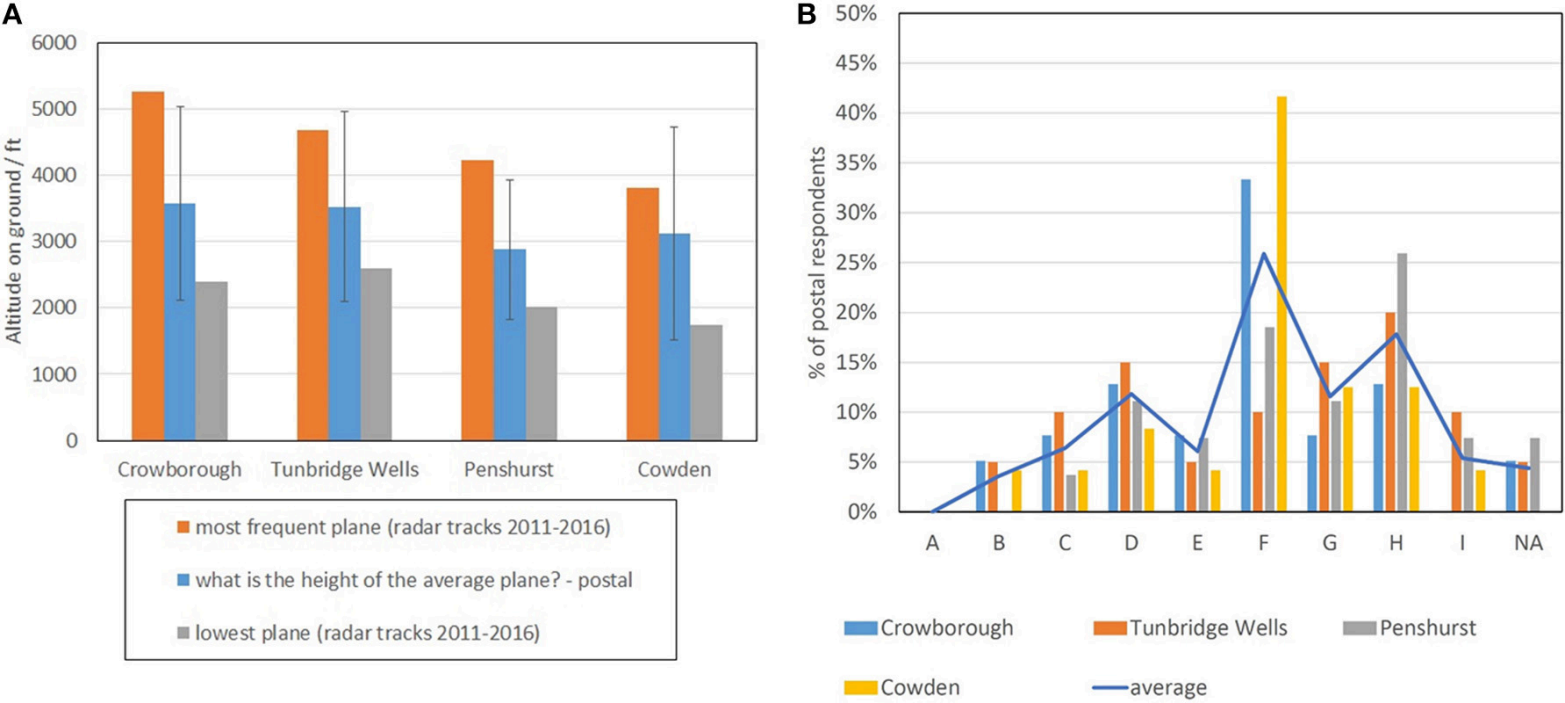
Height **(A)** and size **(B)** of the “average” plane as reported in the received postal questionnaires. Error bars in **(A)** refer to one standard deviation, while letters in **(B)** refer to the size categories described in Figure 4. The “mean” line in **(B)** is mainly a guide to the eye, treating all the survey areas like one single sample.

Figure 5 reports a comparison between the measured heights of the “most frequent” plane (i.e., from Table 1) and the perceived heights of the “average” plane, as reported by the postal respondents in questions C2 and C6 (see Supplementary Data Sheet 1). In looking at these results, it is worth remembering that the wording of the relative questions (see e.g., Figure 2) does not define what the “average” and the “lowest” plane are: these are categories assigned by the respondents according to their perceptions.

Respondents reported a perceived height that was typically lower than the one determined by radar tracks (Table 1). Most of the postal respondents, for instance, (under)estimated the height of the “lowest” plane within 400 ft, while (under)estimating the height of the “most frequent” plane by 900–1,500 ft (see Figure 5A). The fact that the height of lowest plane is so accurately reported highlights its strong presence in the memory of the respondents.

Similarly, most of the respondents reported the correct size class for the lowest plane but perceived the “most frequent” plane to be at least one size larger. According to its size, the “most frequent” plane should in fact be seen in the range C of Figure 4, but only 15% of the respondents judged the “average plane” to be in this class (i.e., first peak from left in Figure 5B). The other respondents reported a size for the “average plane” at least two classes higher.

A plausible reason for this discrepancy (in terms of height and size of the “most frequent” plane) is labeling the postal sample as more prone to negative comments (Janssen et al., 2011). In support to this conclusion, we noted that 22 of the 112 postal respondents (20%) declared to have filed at least one complaint to the airport. These represent about 50% of the highly annoyed in our sample (i.e., a total of 44 out of 112 respondents reported a score ≥7 to the annoyance question D3 in the part regarding “planes”) and 48% of the ones who reported sleep disturbance (i.e., a total of 46 out of 112 respondents scored ≥7 to question D3 in the part for “sleep disturbance”). With the expected percentage of those complaining ranging from 2% (Avery, 1982) to 19% (Van Wiechen et al., 2003) of the highly annoyed ones, this is a much larger value than what reported in other studies (Maziul et al., 2005). This hypothesis was further tested in the field studies, which typically offer a different window into community perceptions.

## Field Interviews

As described above, we decided to run two surveys in parallel to probe both long-term and short-term perceptions. Investigations on outliers or on the correlations between acoustical and visual indicators were only possible by commenting on the planes as they passed over the observer. Running two surveys simultaneously also allows the researchers to maximize community involvement (e.g., picking the age groups or group of respondents not fully represented by the postal survey returns) and, at the same time, build up their own impression of the local reality. In hindsight, we also noticed that sending a postal questionnaire improves the chances of being well-received when visiting for unannounced interviews^6^, just like conducting interviews increases the response rate of postal studies.

To minimize impact on the participants' life, we designed our interviews to last no longer than a successful marketing or fundraising interaction, i.e., 15-min (Market Research Society, 2014). Advantageously, 15-min should also be sufficient to establish a perceptual acoustic judgement, according to recent models of acoustic perception (De Coensel and Botteldooren, 2008) and to some experimental studies on planes (Breugelmans et al et al., 2017) and other traffic sources (Memoli et al., 2008; Memoli and Licitra, 2012).

We assigned to the field interviews also the role of looking at planes “there and then.” This was achieved by what we called “plane spotting”: as soon as a plane appeared in the field of view of the interviewee, the flow of the interview was interrupted, and the interviewer delivered a set of targeted questions related to that specific plane (“single-plane questions”).

### Recruitment

The field interviews in this study occurred unannounced, to avoid the establishment of prejudices that could affect short-term judgements. Consistently, we decided to recruit participants not by appointment, but meeting them on their doorstep or in a local park and to run the interviews in a semi-structured way, to leave more space for free comments and to create a friendlier atmosphere between the researcher and the participant.

In September 2017, the research team visited each survey areas at various times of the day, at least once during the week and once during the weekend. Once in a location, the team split: one was fixed near the noise meter and the other knocked at the doors in a specific road. Then the noise meter was moved in another road and the roles were inverted. Every time one of the researchers encountered a person willing to be interviewed, he/she would start reading the ethics form (see Supplementary Data Sheet 2). In doing so, he/she would formally invite the potential interviewee to be part of the study, would explain our procedure of data storage, would mention how to cancel the responses at any time and would ask for an explicit consent. Following advice from the Ethics Committee at Sussex, we registered consent either by getting a signature or by recording a pre-prepared sentence.

The researcher would then follow the flow suggested by the pre-prepared questionnaire, interrupting it as soon as a plane could be spotted in the sky. In our design, in fact, the goal for each interview was to acquire the interviewee's opinion on at least one passing plane, while the interaction lasted^7^.

### Questionnaire for the Semi-structured Interviews

The guide questionnaire (see Supplementary Data Sheet 2) is like the one used in the postal survey, plus something specific. It has questions on:

- demographics (age, gender, type of home, employment status, local to the area);
- non-acoustical parameters (“feeling in control,” presence of sound insulation at home, sensitivity to unwanted sounds);
- annoyance at home and sleep disturbance (a direct link to the postal questionnaire);
- changes in the number/height/loudness of planes in the past 24 h;
- a section assessing “when do you feel a plane flies over you,” assessed in two questions, like in the postal case.

The key differences with the postal survey are:

- The scales used. Since interactions were verbal, we used in the interviews only 5-points verbal scales throughout (Fields et al., 2001).
- An additional question in the ice-breaking section (i.e., at the start of the guide questionnaire). We asked whether the interviewee had heard about our study. This allows the researchers to identify potential external influences on the interviewee but also, more simply, the interviewees who had already filled in the postal questionnaire.
- The role of outliers was assessed only in the field interviews, interrogating the participant on “extremely noticeable planes” (Questions 8, 9, and 10) and on which of their activities they felt aircraft sounds impacted most.

Whenever a plane passed on sight, however, the interviewer would pass to a “single-plane” questionnaire (inset of the field questionnaire, as shown in Supplementary Data Sheet 2). This part contained questions on the absolute assessment of height/size of the specific plane, but also an assessment of short-term annoyance. The single plane questions also covered by how much the observed aircraft was far from the “average plane.” The reference scales for height (Figure 2) and size (Figure 4) were handed to the participant, so that the researchers could check the appropriate reading distance was used (Supplementary Data Sheet 3).

### Results and Discussion

As in the case of the postal survey, in this work we focus on the perception of height and size as determined during the semi-structured interviews.

#### Demographics

**Figure 6:**
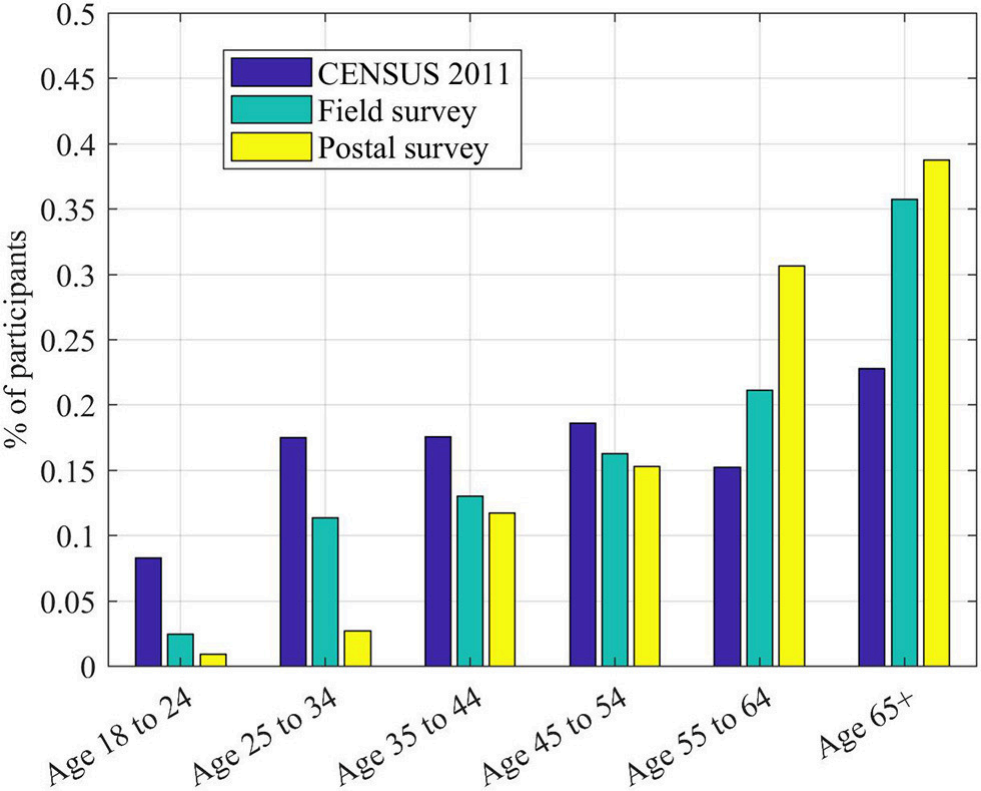
Demographics of the postal and in-person samples, compared to the latest census available for the selected survey areas (Office for National Statistics, 2011). Data reported in this study refer to the Parishes and Wards databases within CENSUS 2011.

In this part of the study, we collected 123 field interviews, observing 242 planes. The questions probing the demographics of the participants (Figure 6), their occupational status and the type of home gave results very similar to the ones in the postal questionnaire. It is worth noting that, while we did not have a direct question on whether the participant worked at the airport, this was part of the conversation: only in one case (i.e., a pilot) the participant declared to be directly related to Gatwick.

#### Perception of Height

**Figure 7:**
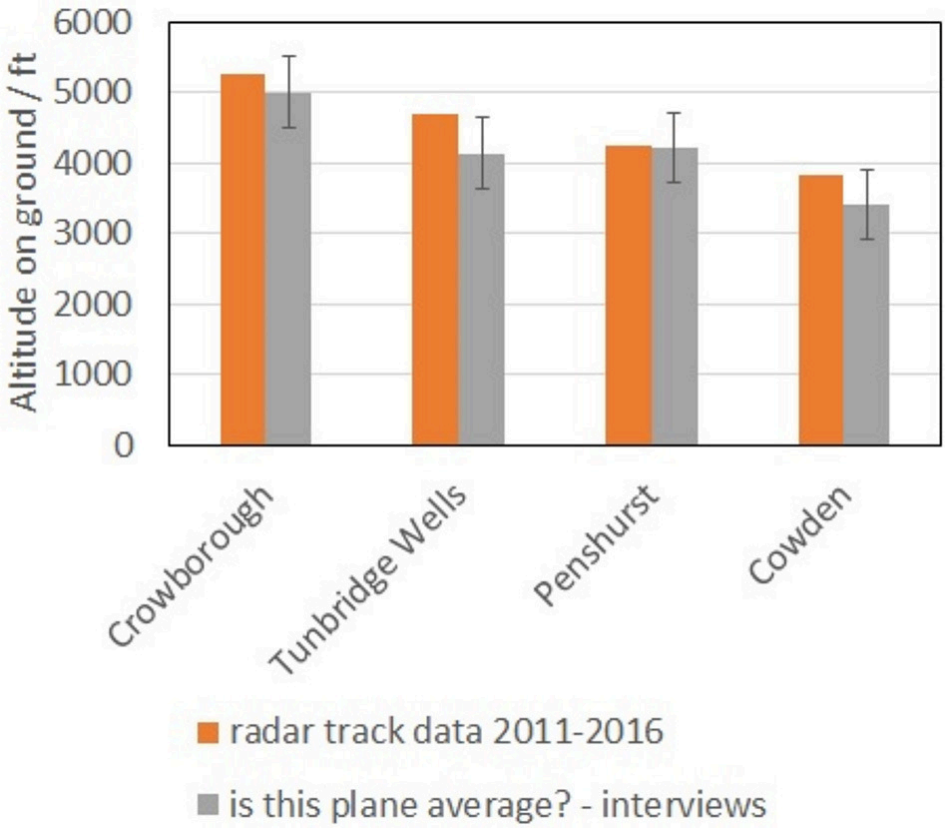
Comparison between the perceived heights of passing planes, as assessed by single-plane questions during field interviews, and the values in Table 1. Data relative to 242 planes out of 242.

Figure 7 reports a comparison between the perceived height of the “average plane,” as determined during interviews, and the height of the “most frequent” plane, from Table 1. The perceived values in Figure 7 were determined by selecting the planes that interviewees labeled as of “average height” and finding the mean and the standard deviation (error bar in Figure 7) of their distribution. This process defines the “average plane.” Figure 7 shows that, in this survey, the “average” plane corresponded, according to our reported answers, to the “most frequent” plane. Also, given the relatively small value of the standard deviation, it can be concluded that interviewees distinguished well when a plane was “average.”

**Figure 8:**
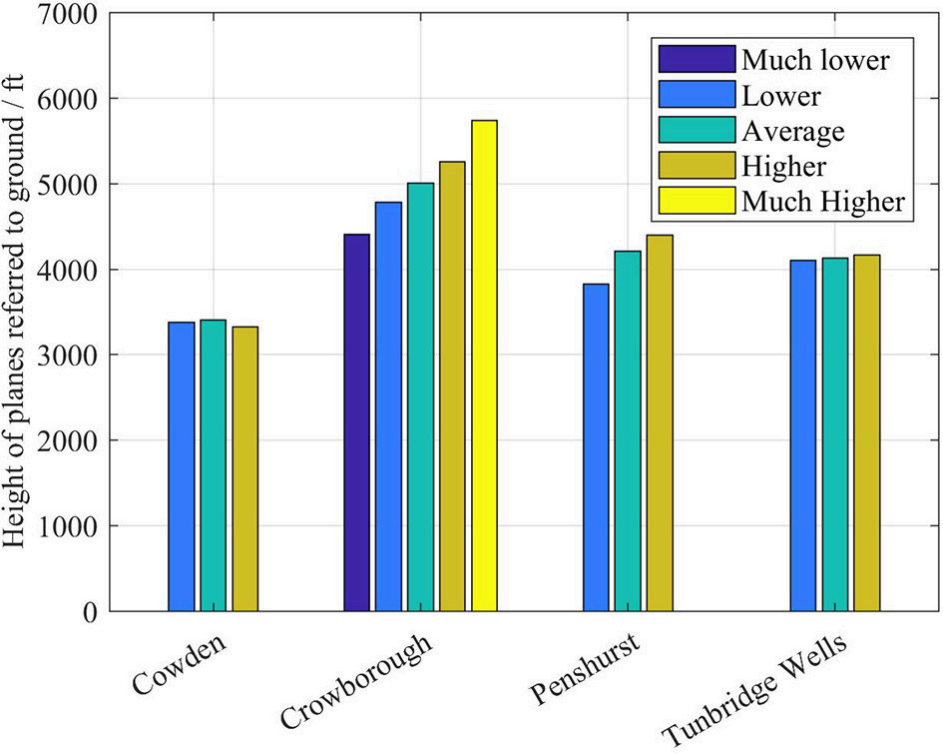
Comparison between the perceived changes from the “average plane” and the real heights of passing planes, as assessed by single-plane questions during field interviews. Data relative to 242 planes out of 242.

Figure 8 shows a comparison between the perceived changes from the “average” plane, as assessed during interviews, and the real changes in height (as determined by radar tracks). Results show that, except for Cowden, interviewees also distinguished well changes from the “average plane”: when planes were higher, they were perceived as higher. Equally, when planes were lower, they were perceived as lower. Particularly interesting is the case of Crowborough, where the planes fly higher than the others and with a wider spread.

**Figure 9:**
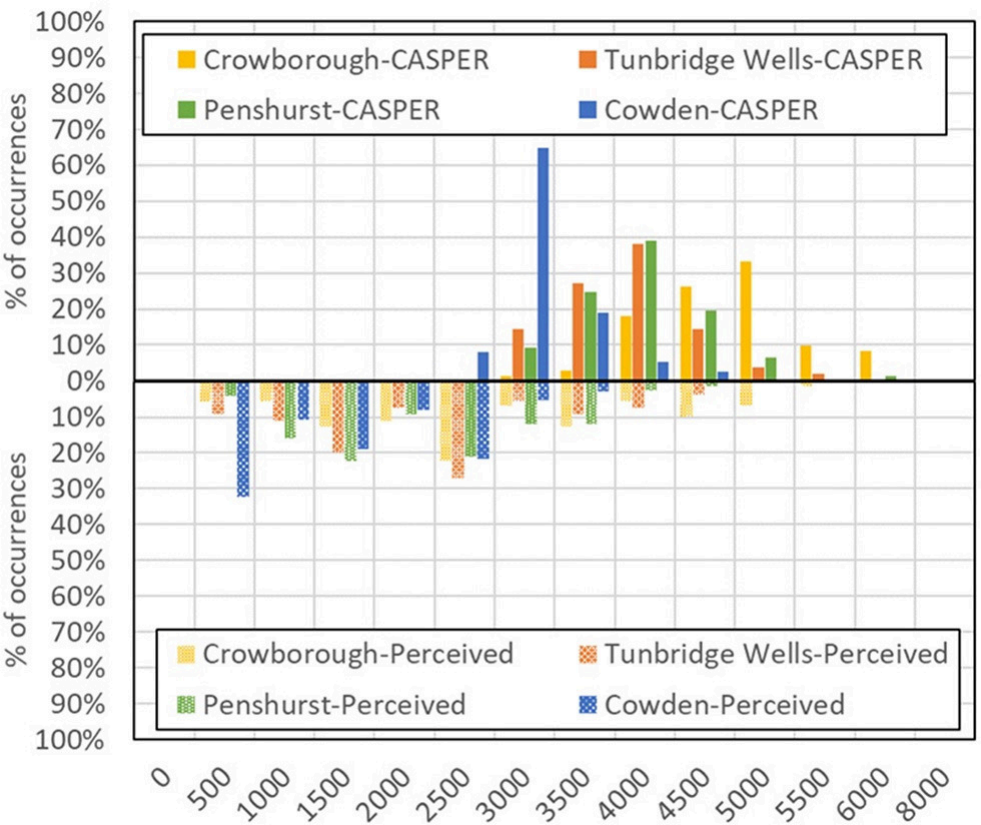
Comparison between the absolute value of the height of a passing plane (from radar tracks, upper part of the graph) and the perceived one, as determined by single-plane questions (lower part of the graph). The label CASPER refers to the app used to track the planes, in post-processing. Data relative to 242 planes out of 242.

Conversely, when asked a numerical judgement on the height of the “average” plane, the interviewees (Figure 9) tended to underestimate it, like the postal respondents (Figure 5A), by about 1,200–1,500 ft (i.e., 350–450 m). As discussed earlier, this is potentially not surprising, given the absence of references on the line of sight between the observer and the plane: it may simply show that the references we used on paper were not sufficient.

Figures 5–8 answer the question “is aircraft height perceived by individuals reasonably accurately,” showing evidence that residents well-know the height of the most frequent plane (i.e., where most of the planes should be in the sky), but also that their absolute estimate of the height of the most frequent plane is not accurate.

Interestingly, the real heights of “most frequent plane” and of the “lowest plane” were within one standard deviation from the perceived height of the “average plane” (this is particularly clear in Figure 5A). There is therefore evidence that, in the process of averaging the height distribution in their memory, postal respondents may have weighted the lowest planes more than the highest ones.

Figures 5–8 also suggest that, since the participants to our study were sensitive to planes not flying like the “average plane” (with a sensitivity that depends on the location, as shown in Figure 8), it is the changes from the average that may trigger negative perceptions and annoyance.

**Figure 10:**
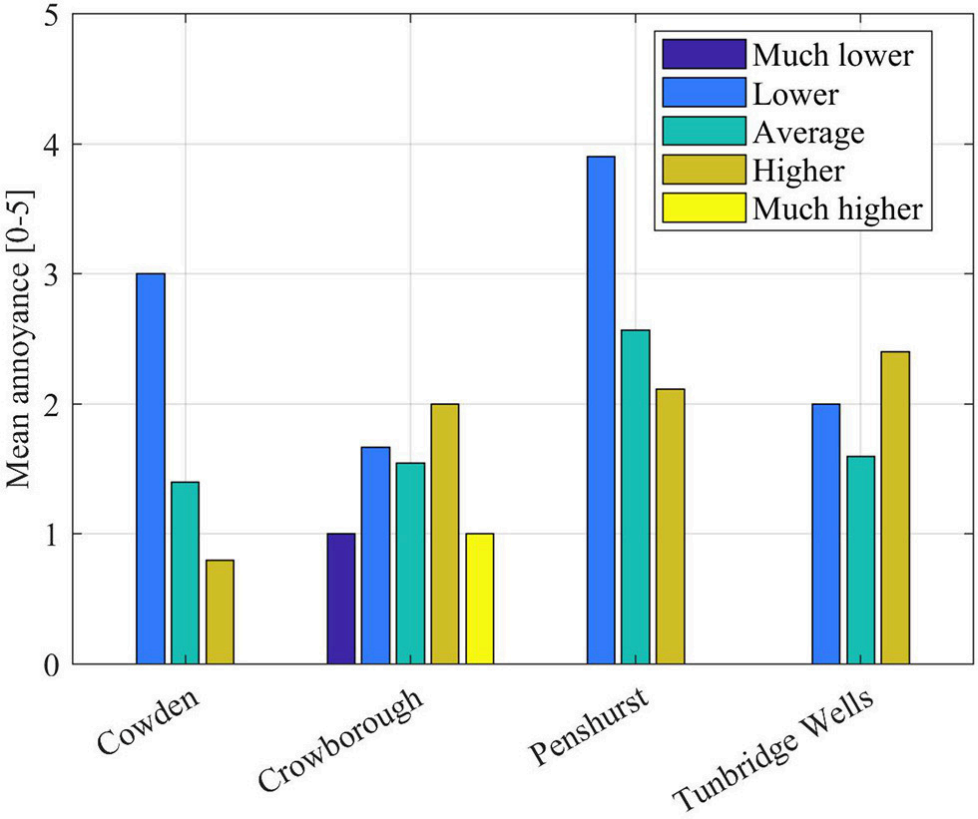
Mean annoyance for different qualitative judgements on the height of a plane, as assessed during “single-plane questions” (242 planes out of 242).

A further evidence in this direction comes from Figure 10, where the mean annoyance (European Environmental Agency, 2010; Guski, 2017) has been calculated relatively to the qualitative judgements on plane height, for each location. Figure 10 shows that, at least for the locations of Penshurst and Cowden, the mean annoyance increases as the planes are perceived to be lower than the “average plane.” The absence of a trend for Crowborough and Tunbridge Wells confirms that a larger sample would need to be analyzed, before drawing definite conclusions.

This finding, however, goes in the direction proposed by a recent study (Filipan et al., 2017), where the authors have found that the perception of tranquil areas in the city parks of Antwerp is mostly affected by the sounds that visitors are not expecting to hear. Changes from the expected may be the cause underpinning annoyance.

#### Perception of Size

**Figure 11:**
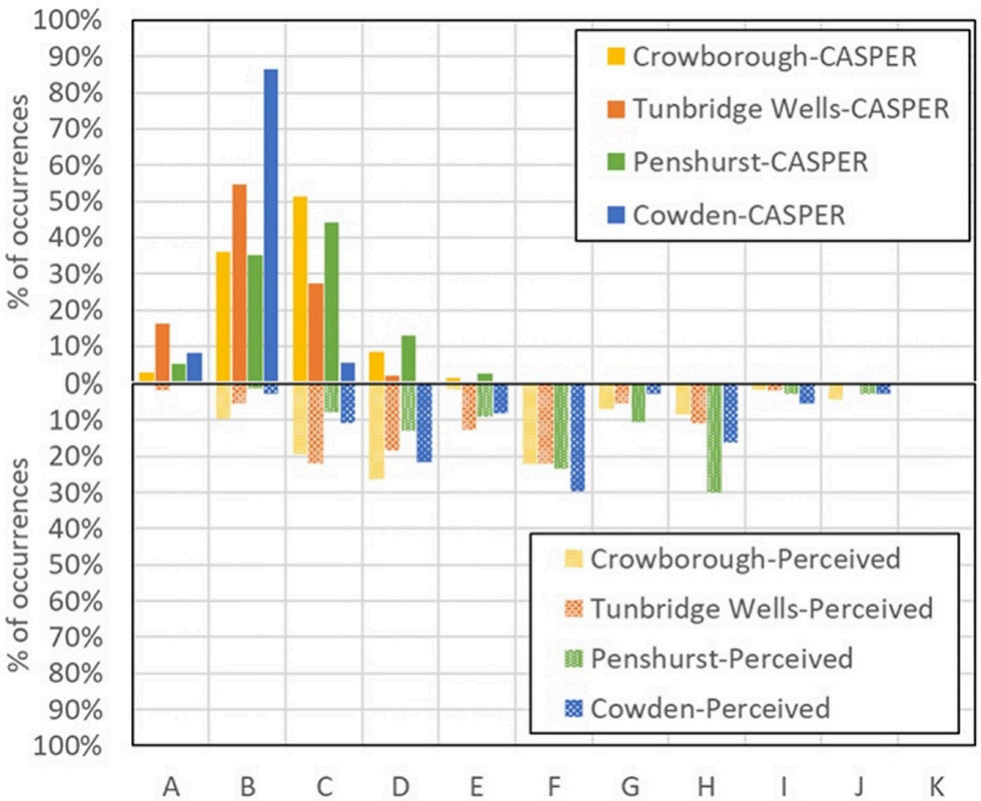
Comparison between the absolute value of the size of a passing plane (from radar tracks, upper part of the graph) and the perceived one, as determined by single-plane questions (lower part of the graph). The label CASPER refers to the app used to determine the actual size of the planes, in post-processing.

If height tends to be underestimated, both surveys confirm that participants tend to overestimate the size of passing planes: as shown in Figure 11 (relative to single-plane observations), they were reported to be up to two classes larger (i.e., up to twice as large). Due to the uncertainty on the reading distance discussed earlier, however, this effect may well be within the limits of the method.

We did not observe any correlation between the error in assessing size (EAS, defined as the ratio between the perceived size and the actual size of a passing plane and therefore reported in arbitrary units or a.u.) and the actual size of a plane (*r* = −0.15, *p* = 0.07). We found instead a correlation between EAS and the visual distance (*r* = 0.66, *p* < 0.001): it is much easier to get the size wrong for planes further away i.e., the size-distance invariance hypothesis fails at large distances, like in the moon illusion (Hershenson, 1989b). Unfortunately, our results do not show a clear trend that could be linked to one of the existing theories for the size-distance paradox (see Supplementary Figure 1).

#### Comparison With Acoustic Indicators

As mentioned earlier (section Characterization of the survey areas), a measurement survey run in parallel to the social surveys: one of its aims was to assign a value of *L*_A, max_ to each passing plane captured during the field interviews. In this part of the study, we only use 144 of the 242 available plane events i.e., those where our tracking procedure managed to assign a unique value of *L*_max_ and were therefore clearly unaffected by other acoustic sources in the background. On these planes we run a preliminary analysis, based on the Pearson correlation test (using MATLAB R18), which did not show any correlation between the error in assessing aircraft height (EAH, defined as the difference between the real height of the plane, as obtained by radar tracks, and the perceived one, as reported during the interviews, with negative values corresponding to underestimation) and the objective variables. Specifically, assuming *p* ≤ 0.05 as significance level, we found no correlation between EAH and the real height (*r* = −0.22, *p* = 0.08), the size of the plane (*r* = 0.045, *p* = 0.56), the visual distance (*r* = 0.16, *p* = 0.06) or the peak noise level during an aircraft pass-by (*r* = −0.11, *p* = 0.178). Recent studies, however, suggest that the Pearson test may not be sufficient while analyzing sparse data (Liu et al., 2012).

**Figure 12:**
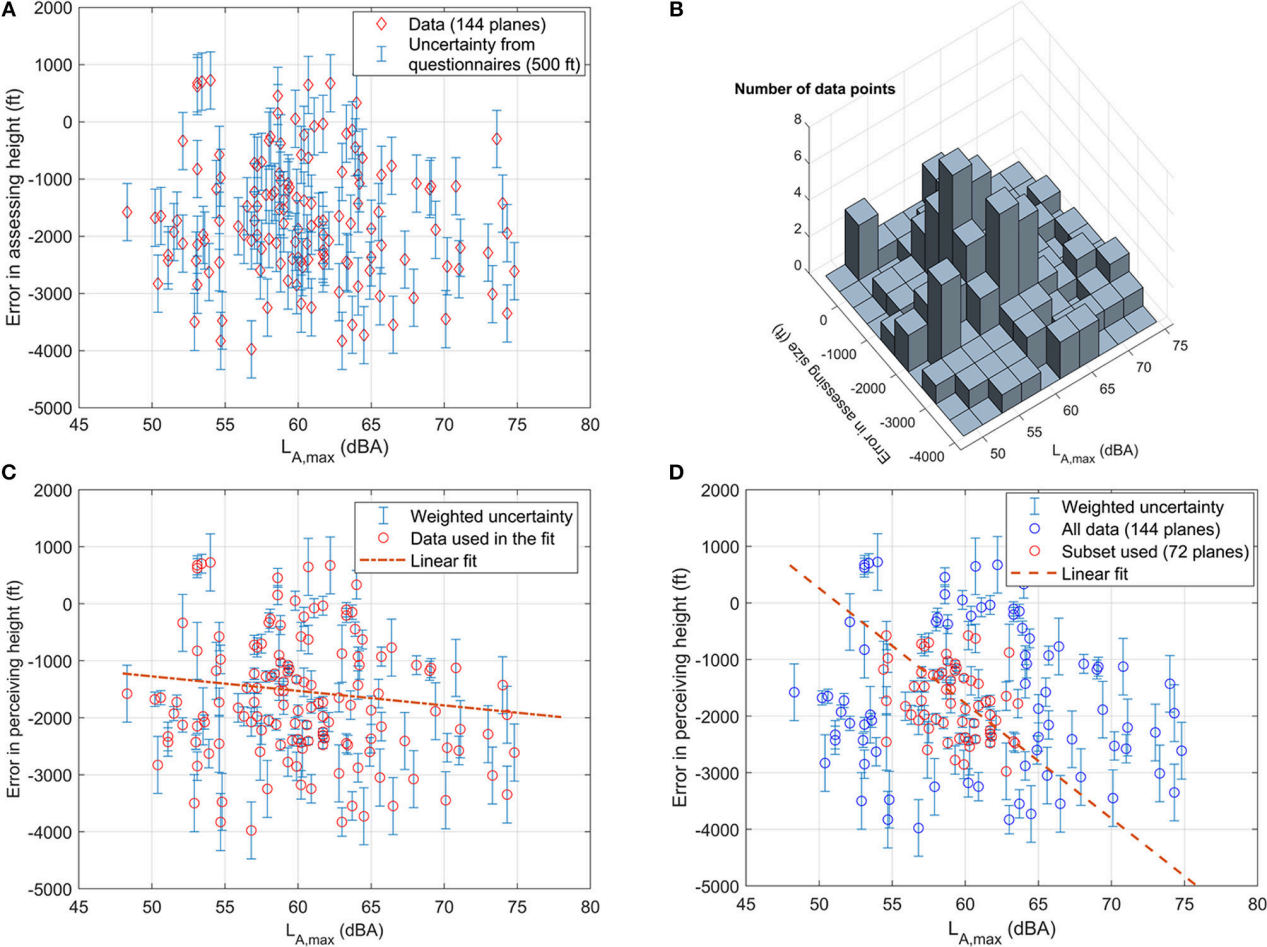
Effect of *L*_*A*, max_ (dBA) on the difference between perceived and real height (ft), as determined by single-plane observations and simultaneous noise measurements: **(A)** all data used for this part of the study (144 planes out of 242); **(B)** the histogram of occurrences; **(C)** fitting the whole dataset with weighted uncertainties; **(D)** fitting the subset of 72 data points obtained by eliminating the data in the regions containing only one or two data points. Error bars in **(A)** are due to the height categories in the questionnaire. Each of **(C,D)** report the corresponding fit.

In the case of EAH vs. *L*_A, max_ (Figure 12A), in fact, while the results are clearly sparse (*SD*:6 *dB* for *L*_A, max_ and 1,000 ft for EAH), most of them can be found in the central region of the graph. This statement is confirmed by Figure 12B, which reports the number of data points in a grid spaced 500 ft vertically and 2 dB horizontally (the pace of the grid reflects the categories in the questionnaire and the measurement uncertainty).

This finding suggests a linear regression *y* = *a*+*b*·*x* based on the chi-square merit function (Press et al., 1992):

(1) $$\begin{array}{r} \chi^{2}\left(a,b\right)=\overset{N}{\underset{i=1}{\sum }}{\left(\frac{y_{i}-a-b\cdot \;x_{i}}{\sigma_{i}}\right)}^{2} \end{array}$$

where *x*_*i*_ is the i-th value of *L*_A, max_, *y*_*i*_ the corresponding value of EAH and σ_*i*_ is the “weighted uncertainty” on the value *EAH*_*i*_, obtained from the initial uncertainty (ε_*i*_ = 500 ft, from the questionnaires) in order to weight some regions of Figure 12A more than others (see below). This method gives *a*±σ_*a*_ and *b*±σ_*b*_ where (Press et al., 1992):

$$\begin{array}{rcl} a & = & \;\frac{\sum_{i}\frac{x_{i}^{2}}{\sigma_{i}^{2}}\cdot \sum_{i}\frac{y_{i}}{\sigma_{i}^{2}}-\sum_{i}\frac{x_{i}}{\sigma_{i}^{2}}\cdot \sum_{i}\frac{x_{i}y_{i}}{\sigma_{i}^{2}}}{\sum_{i}\frac{1}{\sigma_{i}^{2}}\cdot \sum_{i}\frac{x_{i}^{2}}{\sigma_{i}^{2}}\cdot -{\left(\sum_{i}\frac{x_{i}}{\sigma_{i}^{2}}\right)}^{2}}; \end{array}$$

(2) $$\begin{array}{rcl} \;\sigma_{a}^{2} & = & \frac{\sum_{i}\frac{x_{i}^{2}}{\sigma_{i}^{2}}\cdot }{\sum_{i}\frac{1}{\sigma_{i}^{2}}\cdot \sum_{i}\frac{x_{i}^{2}}{\sigma_{i}^{2}}\cdot -{\left(\sum_{i}\frac{x_{i}}{\sigma_{i}^{2}}\right)}^{2}} \end{array}$$

$$\begin{array}{rcl} b & = & \;\frac{\sum_{i}\frac{1}{\sigma_{i}^{2}}\cdot \sum_{i}\frac{x_{i}y_{i}}{\sigma_{i}^{2}}-\sum_{i}\frac{x_{i}}{\sigma_{i}^{2}}\cdot \sum_{i}\frac{y_{i}}{\sigma_{i}^{2}}}{\sum_{i}\frac{1}{\sigma_{i}^{2}}\cdot \sum_{i}\frac{x_{i}^{2}}{\sigma_{i}^{2}}\cdot -{\left(\sum_{i}\frac{x_{i}}{\sigma_{i}^{2}}\right)}^{2}};\; \end{array}$$

(3) $$\begin{array}{rcl} {\;\sigma }_{b}^{2} & = & \frac{\sum_{i}\frac{1}{\sigma_{i}^{2}}}{\sum_{i}\frac{1}{\sigma_{i}^{2}}\cdot \sum_{i}\frac{x_{i}^{2}}{\sigma_{i}^{2}}\cdot -{\left(\sum_{i}\frac{x_{i}}{\sigma_{i}^{2}}\right)}^{2}} \end{array}$$

In this study, the weighted uncertainties σ_*i*_ were assigned to *y*_*i*_ by taking the initial value ε_*i*_ = 500 ft (which is equal for all the points) and dividing it by the number of occurrences in the region that contains *y*_*i*_. Therefore, if (*x*_1_, *y*_1_) and (*x*_2_, *y*_2_) are all the points contained in the same region Ξ of the 2D histogram in Figure 12B, they both get σ_1_ = σ_2_ = 250 ft; if (*x*_3_, *y*_3_) is the only point in region Φ, its uncertainty remains σ_3_ = 500 ft.

This approach corresponds to looking for a regression that does not depend on other parameters, where the single data points have a weight related to their statistical significance (i.e., if a larger number of people gave a similar answer, that answer counts more than others). Using all the data (144 points) and the weights 1/σ_*i*_, minimizing the chi-square functions leads to *a*_1_ = 0±100 ft and *b*_1_ = −26±3 ft/dBA (see Figure 12C). This fit suggests that the louder the plane, the larger the value of EAH. Its “goodness of fit,” however, is barely acceptable: MATLAB *fitnlm* function gives in fact (*r* = −0.149, *p* = 0.07).

We therefore applied a form of subset selection (Miller, 2002), focusing on the center of Figure 12A and neglecting all data with σ_*i*_≥250 ft. In this way only 72 data points (of the 144 available) are used in the fit, but the linear regression is much stronger (*r* = −0.407, *p* < 0.001), with *a*_2_ = 16, 200±600 ft and *b*_2_ = −300±10 ft/dBA in the region 54 ≤ *L*_A, max_ ≤ 64 dBA (see Figure 12D).

To clarify the potential impact of our findings, we will use the fitting line in Figure 12D and consider a plane flying on day 1 over Crowborough at 4,200 ft., with *L*_A, max_ = 57 dBA. Following the vertical at 57 dBA, we encounter the guiding line joining our data at −900 ft., so this plane will be perceived to be flying at 3,300 ft., with *L*_A, max_ = 57 dBA. If the same plane, on day 2, overflies Crowborough at 3,400 ft, its emission as a point source^8^ will increase to *L*_A, max_ = 58.8 dBA. Joining the vertical at 58.8 dBA with the red dotted line gives an increase in the EAH, which becomes ≈−1, 400 ft. The second day, then, this plane would be perceived to fly at 2,000 ft. The plane would be flying lower, by 800 ft., but would be perceived to fly much lower, by ~2,200 ft.

No other correlation was found for EAH, even when the subset selection method was applied to the other variables. If confirmed over a larger sample (e.g., including the 98 plane events not used in this study, as their *L*_A, max_ was affected by non-aircraft sources), these results may give a new insight into the perceptual mechanism causing annoyance due to unwanted plane sounds to rise much quicker (due to changes in perceived height) than the one corresponding to other traffic sources.

**Figure 13:**
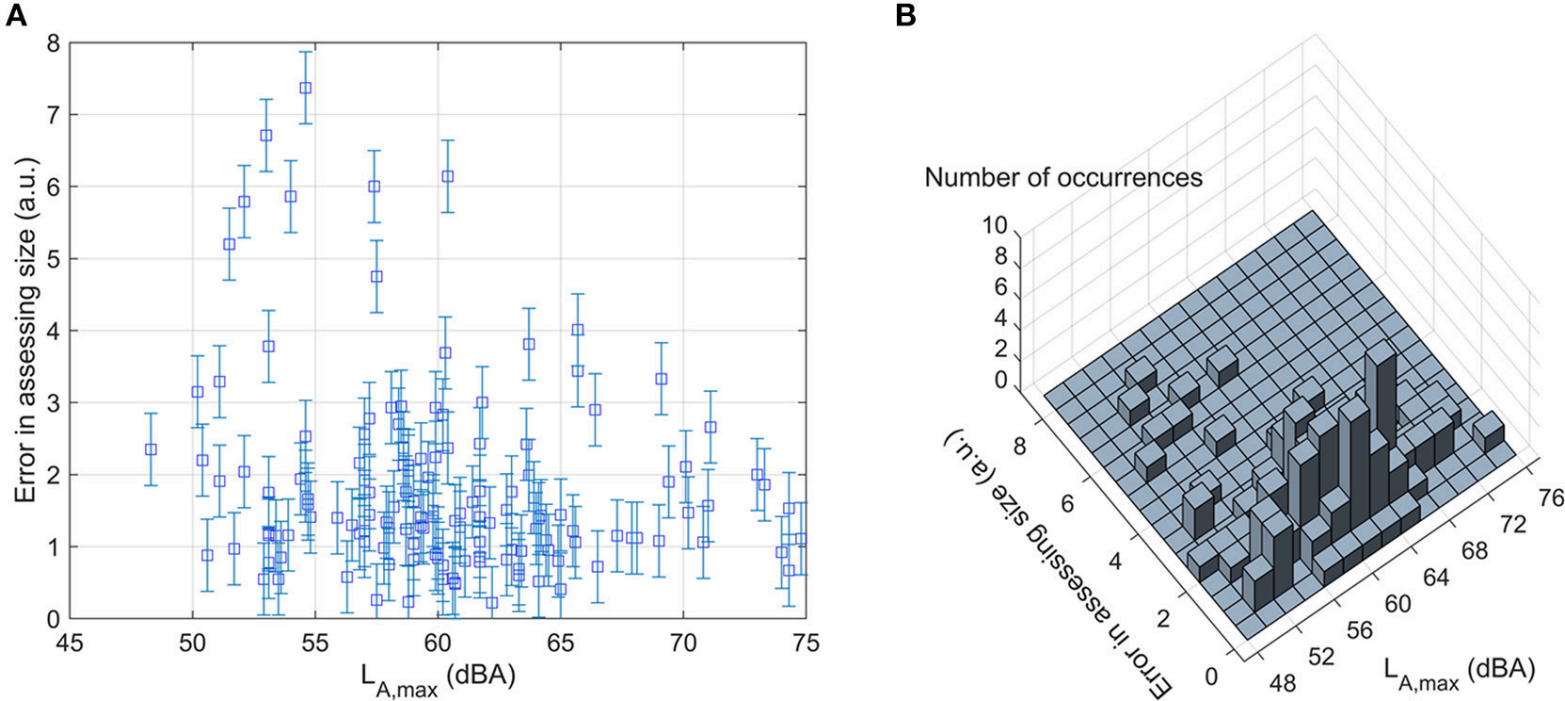
Effect of *L*_*A*, max_ (dBA) on the ratio between perceived and actual size of a plane (EAS), as determined by single-plane observations and simultaneous noise measurements: **(A)** all data used for this part of the study (144 planes out of 242) and **(B)** number of occurrences over a grid of 0.5 (arbitrary units) ×2 (dBA). Error bars represent the pace of the grid (i.e., 0.5 a.u.).

In this study, we could not detect any effect of *L*_A, max_ on the ratio between perceived and actual size (EAS): as shown in Figure 13A, EAS does not depend on *L*_A, max_ (i.e., it stays constant for different values of *L*_A, max_). This conclusion remained similar (*r* = −0.061, *p* = 0.47) even when the subset selection method was applied: as shown in Figure 13B, most of the data clearly align with a horizontal line. Since there is an effect of plane peak emission on perceived height, but not on size, it is reasonable to think that there is no correlation between perceived size and height. This result, if confirmed by a larger sample, may give a negative answer to the question “is there a correlation between aircraft size and height perception?”.

## Overall Discussion

In the previous sections, we have presented the results of testing our method on a selection of 4 survey areas around Gatwick airport. For what concerns the qualitative and the quantitative assessment of height and size, both the postal survey and field survey gave the same result: the main advantage of running two types of survey simultaneously was in reinforcing the confidence in the overall message, even with a limited sample. This consideration is valid in general for studies involving multiple types of social surveys (Bartels et al., 2015; Hiroe et al., 2017).

In some cases, however, our distinct types of survey disagreed: this offered different points of view on the same population and may help inferring the mechanisms underpinning perception in the sampled residents (e.g., whether a perceptual judgement is due to short-term or long-term memories). In our method we put in place a control mechanism to investigate these cases, where postal respondents could volunteer to be interviewed too, but the number of volunteers was eventually very limited (13 over 112). Future studies will need a mechanism to maximize this control sample.

Our proposed method includes 15-min interviews: an absolute minimum in the literature of face-to-face surveys–e.g., (The HYENA Consortium, 2009; Schreckenberg et al., 2016; Civil Aviation Authority, 2017; Hiroe et al., 2017). This choice is extremely convenient and was welcomed by the participants, who only interacted with the researcher for a limited amount of time, but the planned duration was an informed guess, based on previous field studies (e.g., Memoli et al., 2008). A proper psychoacoustic analysis will be needed, before the interview time can be optimized.

In this study, the participants were extremely good at determining where most of the planes should be in the sky (i.e., as the “average” plane was easily identified with the “most frequent” one) but underestimated significantly aircraft height from the ground. We identified a relationship between the noise produced by a plane and this perception error, but nothing similar could be found on the size. We also highlighted a special role of the “lower” planes in the memory of the residents, suggesting a prevalent role of the outliers in perception-based judgement, Our conclusions, however, are limited in their significance by the size of the respondents/interviewees samples: even if the demographics is similar to the local census, future studies will need to be benchmarked on much larger samples.

Our findings support the second of our initial research hypotheses: in absence of clear references, when it can be very difficult to evaluate the absolute height and size of planes passing by, our brain counts on cross-modal interactions between audio and visual stimuli, leading to potentially erroneous judgements on height. The fact that we could not find a correlation between size and height, however, goes against the first hypothesis (i.e., that planes were perceived to be lower because of fleet changes). This may suggest that our brain prefers auditory stimuli to additional visual cues, not only in signal detection (Frassinetti et al., 2002), but also in assessing planes. Specific experiments may be needed for a definite conclusion.

## Conclusions

In this study, we designed a survey method to assess two specific non-acoustical factors in the soundscape perception of residents under the routes of arriving aircraft: the height and the size of arriving planes. The hypothesis of a multisensory interaction between visual and acoustical factors led us to complement existing standardized surveys with specific questions. To our best knowledge, this approach, used in the past for soundscape assessment, has not been applied to aircrafts before.

The ambiguity on whether height effects on perception were due to long-term memory or short-term judgements, and the desire to maximize the involvement of residents, led us to design two different interaction modalities, to be run in parallel: a 40-min long questionnaire and a 15-min interview. The first, delivered by post, was designed to be completed by the participants unassisted, presumably indoors. The second was designed to be run with a researcher, who would recruit the participant either on their doorstep or in a local park, for interviewing him/her outdoors. Interviews also included a component of “plane spotting,” which was used to assess perceptual judgements “there and then.”

Our “double-survey” method, assisted by acoustic measurements and aircraft tracking, was tested in 4 locations around Gatwick airport in the summer of 2017, involving a total of ~200 participants.

When the two surveys arrived at a similar result, the outcome message was reinforced. In this way, we found evidence that:

a. Participants living below arriving aircraft could correctly describe the “average plane” i.e., the most frequent aircraft in their area.
b. Qualitatively, participants were very good at accurately perceiving how a passing aircraft was different from the “average plane”: in height, size, and distance from where they lived.
c. Quantitatively, most participants underestimated the height of a specific aircraft—including the “average” one—by between 1,200 and 1,500 ft and overestimated its size by as much as twice.
d. For the same height, louder planes are perceived as lower, but not as larger.
e. Planes which are different from the “average plane” (i.e., “outliers”) are the ones affecting perception, generating annoyance.

These observations, if confirmed in other studies or with a larger sample, may underpin the differences between the perception of arriving aircraft and the annoyance judgements on other sources of noise (i.e., unwanted sounds). Assessing the visual variations in the height of arriving planes may become one of the key non-acoustical factors in surveys oriented to arriving aircraft. The fact that outliers seem to play a key role in the perception of overflown residents, even more than the absolute height of the “most frequent plane,” may have a significant impact on aircraft movement strategies in the future.

The fact that the two parallel surveys captured the impressions of two complementary parts of the population, if confirmed in other studies, may affect the way we determine community perception in the future: running two types of samples, supported by measurements, may become the new standard.

## Ethics Statement

This study was carried out in accordance with the recommendations of Data Protection Act 1998 and the General Data Protection Regulation (GDPR). The protocol was approved on 23/08/2017 by the Sciences & Technology Cross-Schools Research Ethics Committee at the University of Sussex, under project reference number ER/GM330/1. All subjects gave written or recorded informed consent, in accordance with the Declaration of Helsinki.

## Author Contributions

GM led the study, conducted data analysis and wrote the paper. GH-F participated to the survey design and to the field survey. SM chaired the steering committee at Gatwick: he designed the scoping document and provided information on the context.

### Conflict of Interest Statement

The authors declare that the research was conducted in the absence of any commercial or financial relationships that could be construed as a potential conflict of interest.

## Footnotes

^1^In this study, “visual distance” is the distance between the eye of the observer and a passing plane, along the line connecting the two. For clarity, the closest distance to the observer was considered, both during the interviews and in post-processing.

^2^An assumption very close to the real facts, as shown from the distributions acquired by radar tracks for the summers in the period 2011-2016.

^3^We only had measurements for 21 days during the peak summer period, so our values of *L*_*DEN*_ are not yearly averages.

^4^e.g. “I didn't notice the plane, but now that you mention it, it is annoying”. As in quantum mechanics, the act of measuring (perception) influences the result.

^5^The A330 was chosen as reference since it is the closest in size to the mean of all planes that arrive at Gatwick, and thus should produce the least amount of error.

^6^Randomly selected residents who have already received the postal questionnaire know already what is happening, while others may have heard by word-of-mouth.

^7^Preliminary tests, conducted with students before going in the field, showed that 15 minutes allowed a maximum of three planes to be observed for each participant.

^8^i.e. assuming a decrease in *L*_A, max_ of 6 dBA for each doubling of the distance, which was confirmed by the measurements.
