# Supplementary material for: Soundscape Assessment of Aircraft Height and Size
Source: Front Psychol. 2018 Dec 18;9:2492. doi: 10.3389/fpsyg.2018.02492 (PMC6305372; doi:10.3389/fpsyg.2018.02492)
Supplement: Supplementary file 1 [file Data_Sheet_1.PDF]

Participant ID_______________

POSTAL QUESTIONNAIRE

Following the introductory letter

Participant ID_______________

Your views on the local environment

Answers given should reflect the **views of one person (18+ years old)**, ideally the most familiar with daily noise. Skip any questions that do not apply to you. In returning this questionnaire, you allow the University of Sussex to use the data for this study.

Some initial questions

1. Have you answered a noise survey before? ❑ Yes ❑ No
2. Has anyone in your home made an official noise complaint? ❑ Yes ❑ No
3. What is today's date? (DD/MM/YYYY) ______ / ______ / 2017

SECTION A - Yourself

A1. How old are you? I am ______ years old

A2. What gender are you? ___________

A3. What is your current employment status? (Tick all that apply)

❑ Full-time employed ❑ Full-time student ❑ Retired
❑ Part-time employed ❑ Part-time student ❑ Unemployed
❑ Self-employed ❑ Working from home ❑ Carer (home / family)
❑ Would rather not say ❑ Other: _____________ (please state)

SECTION B - Your circumstances

B1. How long have you lived in **this home**? _______ Year(s) _______ Month(s)

B2. How long have you lived in this **village / town**? _______ Year(s) _______ Month(s)

B3. **About this area** — Definitely dislike (0) … Neither like, Nor dislike … Definitely like (10)

Question	0	1	2	3	4	5	6	7	8	9	10
How much do you **like** living in this area?	☐	☐	☐	☐	☐	☐	☐	☐	☐	☐	☐

B4. What **type of home** do you currently live in?

❑ House: detached ❑ House: semi-detached ❑ House: mid-terrace
❑ Bungalow: detached ❑ Bungalow: semi-detached ❑ Bungalow: mid-terrace
❑ Flat ❑ Maisonette (Flat on 2+ floors) ❑ Other: ____________

B5. Do you have any form of **noise insulation** at home? (Tick all that apply)

❑ Windows (double-glazing) ❑ Roof insulation ❑ Don't know
❑ Windows (triple-glazing) ❑ Wall insulation ❑ Other: ____________

B6. Was any **noise insulation** applied in the **last 12 months**? (Tick all that apply)

❑ No ❑ Yes - Windows ❑ Yes - Roof ❑ Yes - Walls ❑ Don't know

Participant ID_______________

B7. When you are indoors, at which times are you aware of noise outside your home?

**(Tick all that apply)**	Day (7AM-7PM)	Evening (7PM-11PM)	Night (11PM-7AM)
Mon-Fri	❑	❑	❑
Sat-Sun	❑	❑	❑

B8. **Control**

No control — Some control — Complete control

Question	0	1	2	3	4	5	6	7	8	9	10
When indoors, how much **control** do you have over the impact of external noise (e.g. shutting windows)?	☐	☐	☐	☐	☐	☐	☐	☐	☐	☐	☐

SECTION C - PLANE HEIGHT

In this section, we would like you to **guess** some heights of planes when they are nearest to your home (see dotted line below).

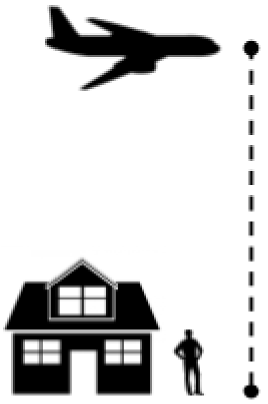

For Reference (not to scale)

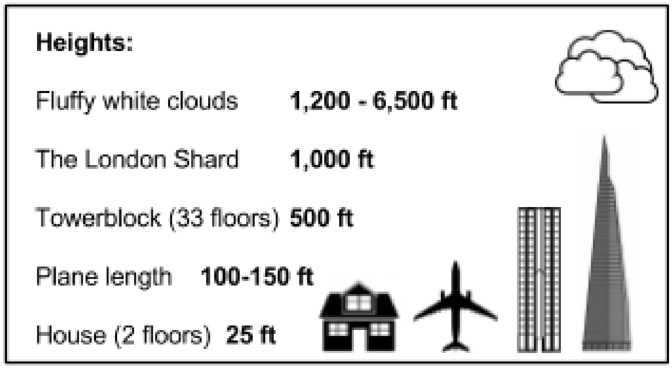

C1. I would **guess** that last month the **lowest** plane was at about:

❑ 500 ft ❑ 1,000 ft ❑ 1,500 ft ❑ 2,000 ft ❑ 2,500 ft ❑ 3,000 ft
❑ 3,500 ft ❑ 4,000 ft ❑ 4,500 ft ❑ 5,000 ft ❑ 5,500 ft ❑ 6,000 ft

C2. I would **guess** that last month the **average** plane was at about:

❑ 500 ft ❑ 1,000 ft ❑ 1,500 ft ❑ 2,000 ft ❑ 2,500 ft ❑ 3,000 ft
❑ 3,500 ft ❑ 4,000 ft ❑ 4,500 ft ❑ 5,000 ft ❑ 5,500 ft ❑ 6,000 ft

C3. **Planes flying over**

None of the flights — Half the flights — All the flights

Question	0	1	2	3	4	5	6	7	8	9	10
Of the flights you notice, what proportion **fly over** you?	☐	☐	☐	☐	☐	☐	☐	☐	☐	☐	☐

Participant ID_______________

C4. Tick all the boxes where you would consider the plane to be **flying over** you

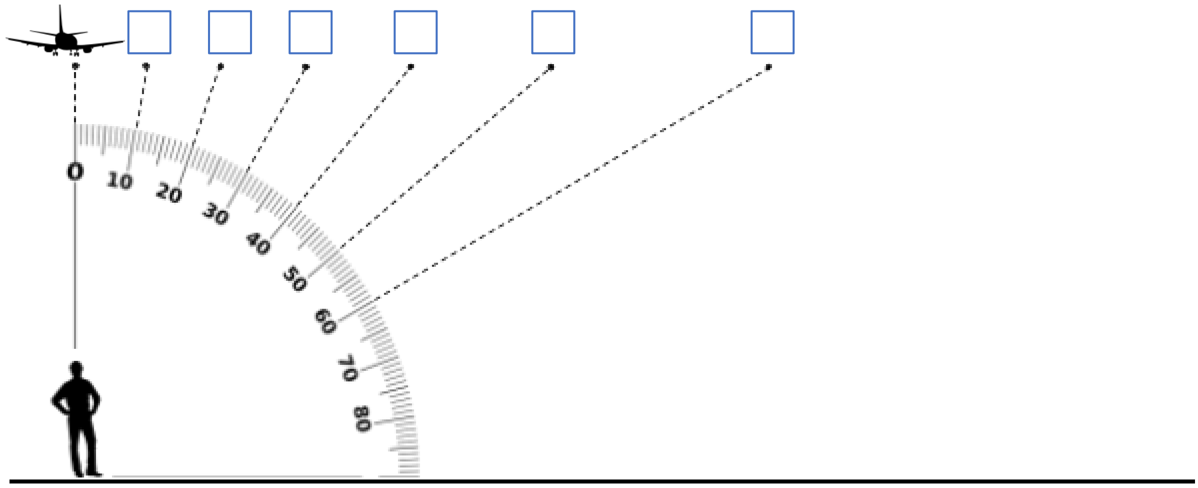

When holding this page at a **normal reading distance** (about 45cm away), which of the below silhouettes are about the size of planes flying near your home?

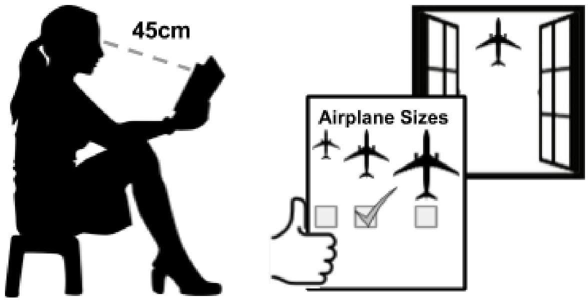

C5. Which silhouette below looks about the size of the **lowest** plane flying over your home?

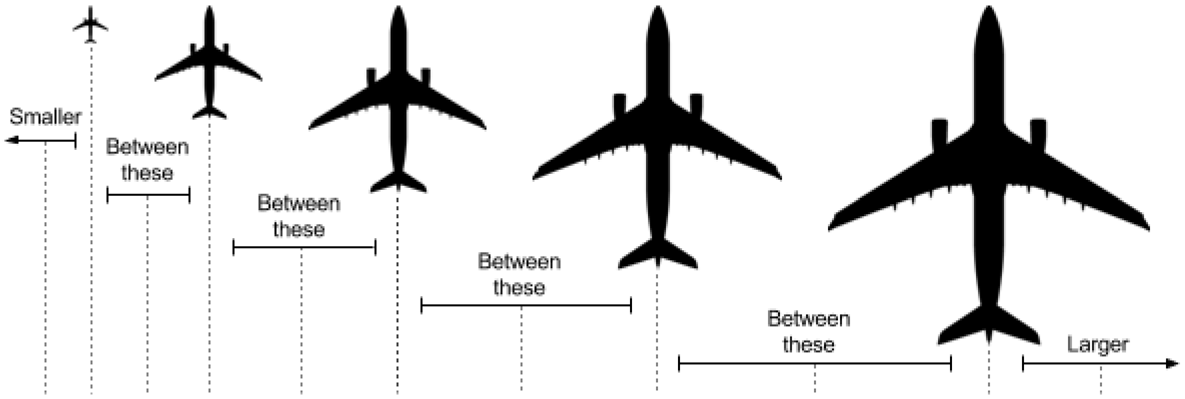

C6. Which silhouette looks the size of a plane flying at **average height** above your home?

C7. **Physical size** of planes

Much smaller — The same size — Much larger

Question	0	1	2	3	4	5	6	7	8	9	10
Compared to **a year ago**, the **average** plane now is...											
Compared to **5 years ago**, the **average** plane now is...											

Participant ID_______________

C8. Plane **height**

In the **last month**, I would describe...	0 (Extremely low)	1	2	3	4	5 (Neither low, nor high)	6	7	8	9	10 (Extremely high)
the **lowest** plane that **flew over me** as	☐	☐	☐	☐	☐	☐	☐	☐	☐	☐	☐
the **average** plane that **flew over me** as	☐	☐	☐	☐	☐	☐	☐	☐	☐	☐	☐

C9. Plane **height changes**

Question	0 (Got much lower)	1	2	3	4	5 (Stayed the same)	6	7	8	9	10 (Got much higher)
Over the **last year,** the **lowest** plane flying over me has	☐	☐	☐	☐	☐	☐	☐	☐	☐	☐	☐
Over **last 5 years**, the **lowest** plane flying over me has	☐	☐	☐	☐	☐	☐	☐	☐	☐	☐	☐
Over the **last year**, the **average** plane flying over me has	☐	☐	☐	☐	☐	☐	☐	☐	☐	☐	☐
Over **last 5 years**, the **average** plane flying over me has	☐	☐	☐	☐	☐	☐	☐	☐	☐	☐	☐

C10. **Amount** of planes

Question		0 (Significantly decreased)	1	2	3	4	5 (Stayed the same)	6	7	8	9	10 (Significantly increased)
Over the **last year**, the number of **planes**...	between 7AM and 7PM has	☐	☐	☐	☐	☐	☐	☐	☐	☐	☐	☐
	between 7PM and 11PM has	☐	☐	☐	☐	☐	☐	☐	☐	☐	☐	☐
	between 11PM and 7AM has	☐	☐	☐	☐	☐	☐	☐	☐	☐	☐	☐
Over the **last 5 years**, the number of **planes**...	between 7AM and 7PM has	☐	☐	☐	☐	☐	☐	☐	☐	☐	☐	☐
	between 7PM and 11PM has	☐	☐	☐	☐	☐	☐	☐	☐	☐	☐	☐
	between 11PM and 7AM has	☐	☐	☐	☐	☐	☐	☐	☐	☐	☐	☐

C11. **Noticing** noise

Question	0 (Much less noticeable)	1	2	3	4	5 (About the same)	6	7	8	9	10 (Much more noticeable)
Is plane noise **less** or **more** noticeable than all other noises (traffic, neighbours, animals etc)?	☐	☐	☐	☐	☐	☐	☐	☐	☐	☐	☐

Participant ID_______________

SECTION D - Noise over the past 12 months

All questions below refer to when you have been **inside your home** (i.e., indoors) over the **past 12 months** (or however many months you have lived at your current home).

D1. **All sounds**

Completely dislike — Neither like, nor dislike — Completely like

Question	0	1	2	3	4	5	6	7	8	9	10
When indoors, how much do you **like** the typical <u>sounds</u> that come from outside your home?											

D2. **All noise**

Not at all — Moderately — A lot

Question	0	1	2	3	4	5	6	7	8	9	10
When indoors, how much has <u>all noise</u> from outside your home **bothered**, **disturbed** or **annoyed** you?											

D3. How much have the following **types of noise** bothered, disturbed or annoyed you?

Not at all — Moderately — A lot

Noise	Question	0	1	2	3	4	5	6	7	8	9	10
Plane Noise 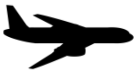	How much has this **bothered, disturbed,** or **annoyed** you?											
	How much has this **disturbed your sleep**?											
	How much has this **spoiled** your homelife?											
Traffic Noise 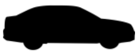	How much has this **bothered, disturbed,** or **annoyed** you?											
	How much has this **disturbed your sleep**?											
	How much has this **spoiled** your homelife?											
All Other Noises 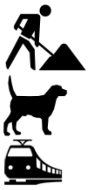	How much have these **bothered, disturbed,** or **annoyed** you?											
	How much have these **disturbed your sleep**?											
	How much have these **spoiled** your homelife?											

Participant ID_______________

D4. Does **noise from planes** disturb any of the following activities for you?

Not at all | Sometimes | All the time

Noise	Activity	0	1	2	3	4	5	6	7	8	9	10
Noise from planes 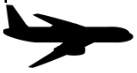	This disturbs my **conversations** indoors (inc. phone calls, online)											
	This disturbs my **TV viewing** (including radio, games, music)											
	This disturbs my **reading** or **concentration** indoors											
	This disturbs my **working** indoors											
	This disturbs my **relaxing** indoors											
	Disturbs my **family** or **pets**											
	Prevents me **opening windows**											
	Stops me enjoying the **outside**											

D5. Do **all other types of noise** (i.e. not planes) disturb any of the following activities?

Not at all | Sometimes | All the time

Noise	Activity	0	1	2	3	4	5	6	7	8	9	10
Other types of noise (except planes) 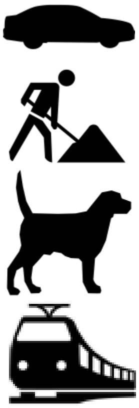	This disturbs my **conversations** indoors (inc. phone calls, online)											
	This disturbs my **TV viewing** (including radio, games, music)											
	This disturbs my **reading** or **concentration** indoors											
	This disturbs my **working** indoors											
	This disturbs my **relaxing** indoors											
	Disturbs my **family** or **pets**											
	Prevents me **opening windows**											
	Stops me enjoying the **outside**											

Participant ID_______________

D7. Indicate how much you **agree** or **disagree** with the following statements.

Strongly disagree (0) — Neither agree, nor disagree (5) — Strongly agree (10)

Question	0	1	2	3	4	5	6	7	8	9	10
I wouldn't mind living on a noisy street if the apartment I had was nice.	☐	☐	☐	☐	☐	☐	☐	☐	☐	☐	☐
I am more aware of noise than I used to be.	☐	☐	☐	☐	☐	☐	☐	☐	☐	☐	☐
No one should mind much if someone turns up his stereo full blast once in awhile.	☐	☐	☐	☐	☐	☐	☐	☐	☐	☐	☐
At movies, whispering and crinkling sweet wrappers disturb me.	☐	☐	☐	☐	☐	☐	☐	☐	☐	☐	☐
I am easily awakened by noise.	☐	☐	☐	☐	☐	☐	☐	☐	☐	☐	☐
If it's noisy where I'm studying, I try to close the door or window or move someplace else.	☐	☐	☐	☐	☐	☐	☐	☐	☐	☐	☐
I get used to most noises without much difficulty.	☐	☐	☐	☐	☐	☐	☐	☐	☐	☐	☐
It would not matter to me if an apartment I wanted to rent was located across from a fire station.	☐	☐	☐	☐	☐	☐	☐	☐	☐	☐	☐
Even music I normally like will bother me if I'm trying to concentrate.	☐	☐	☐	☐	☐	☐	☐	☐	☐	☐	☐
It wouldn't bother me to hear the sounds of everyday living from my neighbours (footsteps, running water, etc).	☐	☐	☐	☐	☐	☐	☐	☐	☐	☐	☐
When I want to be alone, it disturbs me to hear outside noises.	☐	☐	☐	☐	☐	☐	☐	☐	☐	☐	☐
I'm good at concentrating no matter what is going on around me.	☐	☐	☐	☐	☐	☐	☐	☐	☐	☐	☐
In a library, I don't mind if people carry on a conversation if they do it quietly.	☐	☐	☐	☐	☐	☐	☐	☐	☐	☐	☐
There are often times when I want complete silence.	☐	☐	☐	☐	☐	☐	☐	☐	☐	☐	☐
Motorcycles ought to be required to have bigger mufflers.	☐	☐	☐	☐	☐	☐	☐	☐	☐	☐	☐
I find it hard to relax in a place that's noisy.	☐	☐	☐	☐	☐	☐	☐	☐	☐	☐	☐
I get mad at people who make noise that keeps me from falling asleep or getting work done.	☐	☐	☐	☐	☐	☐	☐	☐	☐	☐	☐
I wouldn't mind living in an apartment with thin walls.	☐	☐	☐	☐	☐	☐	☐	☐	☐	☐	☐
I am sensitive to noise.	☐	☐	☐	☐	☐	☐	☐	☐	☐	☐	☐

Participant ID_______________

Permission for the treatment of data

Thank you for completing the questionnaire! This study has been approved by the Sciences & Technology Cross-Schools Research Ethics Committee (crecscitec@sussex.ac.uk). The project reference number is ER/GM330/1 and the University of Sussex has insurance in place to cover its legal liabilities in respect of this study. By returning the questionnaire, you give the research team the permission to treat your responses anonymously. If you would like us to link your answers to your specific location, please enter your postcode below:

Postcode: _______________

If you desire to remove your answers from our study at a later stage, please let us know by contacting us at the email address noise.survey@sussex.ac.uk before the 1st of October 2017. After this date, removal will no longer be possible.

Feedback to us (University of Sussex)

Below we would like to give you the opportunity to express the views of yourself and the rest of your household that have not been captured so far.

- **Do you feel the questionnaire accurately captured your views?**
- **Were there any question responses you would like to elaborate on here?**

❏ Tick if you are happy for us to include your feedback in our final report

❏ Tick if you are happy for us to include your data again in future studies if these have gained independent ethically approval, based on the strict confidentiality terms described before.

-----------------The research team will tear this part to separate personal data from questionnaire answers--------------------

Interview Appointment Schedule (leave blank if you do not wish us to contact you)

I authorize you to contact me to arrange an interview for this study. I understand that only a random selection of interviews will be eventually conducted. My personal data will be kept separated from my answers above unless I give explicit written consent (e.g., during the interview), will only be kept for the duration of this study and will not be shared with anyone else.

Name: ____

Address: ____ Postcode: ____________

Phone: ___________________________ E-mail: _______________________________

It is best to contact me via: ❏ Phone ❏ E-mail

Best time to contact me: ❏ Morning ❏ Afternoon ❏ Evening

Signed: ____________________________________ Date: ____________________
