# Supplementary material for: Soundscape Assessment of Aircraft Height and Size
Source: Front Psychol. 2018 Dec 18;9:2492. doi: 10.3389/fpsyg.2018.02492 (PMC6305372; doi:10.3389/fpsyg.2018.02492)
Supplement: Supplementary file 2 [file Data_Sheet_2.PDF]

IN PERSON QUESTIONNAIRE and CONSENT SHEET

For further information about this research please contact Dr Giles Hamilton-Fletcher or the project supervisor Dr Gianluca Memoli at noise.survey@sussex.ac.uk. This study has been approved by the Sciences & Technology Cross-Schools Research Ethics Committee (crecscitec@sussex.ac.uk). The project reference number is ER/GM330/1. If you have any ethical concerns, please contact the ethics chair or project supervisor. University of Sussex has insurance in place to cover its legal liabilities in respect of this study.

Study Name: In person noise survey
Date: 15/08/2017 Participant ID____________
Version: 1

INFORMATION SHEET

Invitation to take part

You are being invited to take part in a quick face-to-face interview on the perceived height of planes. The purpose of the study is to understand the relationship between measured heights of planes, perceived heights and impact on local communities. We would like to thank you for carefully reading this information sheet, a copy of which you can keep for your records. This study is being conducted by Dr Giles Hamilton-Fletcher, Dr Gianluca Memoli and Mr Federigo Ceraudo, all from the School of Engineering and Informatics at the University of Sussex. They are happy to be contacted at noise.survey@sussex.ac.uk if you have any questions. Even if the cost of this study has been met by Gatwick Airport Limited, the work is being carried out independently by the University of Sussex.

Why have I been invited for testing and what will I do?

We are interviewing approximately 90 adults across 3 locations underneath the westerly arrivals flight path for Gatwick Airport. The interviews take under 10 minutes to complete, and follow a repeated list of questions. During the interview we will ask you about your experiences with various sources of noise in the environment and to make some judgements about planes in the sky. In parallel, we will be monitoring acoustic indicators and noting the identity of planes passing above us, looking for correlations between your answers and these measurements.

What will happen to the results and my personal information?

The results of this research will be written into a public report and may appear in scientific publications on local noise perception. We anticipate being able to provide a summary of our findings on request from 01/01/2018 (noise.survey@sussex.ac.uk). Your anonymity will be ensured at all stages, in the way described in the consent information below. Please read this information carefully and then, if you wish to take part, we will either require recorded verbal consent or a signature to show you have fully understood this sheet, and that you consent to take part in the study as it is described here.

Study Name: In person noise survey
Date: 15/08/2017 Participant ID___________
Version: 1

CONSENT (COPY FOR THE PARTICIPANT)

- I understand that by giving verbal or written consent below I am agreeing to take part in this University of Sussex research, and that I have read and understood this information sheet.

- I understand that my participation is entirely voluntary, that I can choose not to participate in part or all of the study, and that I can withdraw at any stage of testing without having to give a reason and without being penalised in any way.

- I understand I can request without penalty that my data be withdrawn and deleted even after testing is complete, any time up until the results are analysed (01/10/2017). I will contact you at noise.survey@sussex.ac.uk if I desire to do so, quoting the questionnaire ID at the top of the page.

- I consent to the processing of my personal information provided (age, gender etc) for the purposes of this research study. I understand that such information will be treated as strictly confidential (subject to legal limitations) and handled in accordance with the Data Protection Act 1998.

- I understand that my collected data will be stored in a de-identified way (e.g. using ID numbers not names), and kept separate from other details about me (e.g. from the consent form). Electronic data will be stored on a password-protected computer, and hard-copies will be stored behind a locked door. De-identified data may be made publicly available through 'Sussex Research Online' online data repository alongside publications.

- I understand that my identity will remain confidential in any written reports of this research, and that no information I disclose will lead to the identification in those reports of any individual either by the researchers or by any other party, without first obtaining my written permission.

- I understand that my name and data will not be shared with any third party outside the research group, unless I later provide written permission.

❑ Tick here if you are providing a recorded consent

Read **"My name is ___________, my participant ID is _______________ and I consent to be part of this study."**

❑ Tick here if you are providing a written consent

______________________ _______________ ____________________

Name of Participant Date Signature

For further information about this research please contact Dr Giles Hamilton-Fletcher or the project supervisor Dr Gianluca Memoli at noise.survey@sussex.ac.uk. This study has been approved by the Sciences & Technology Cross-Schools Research Ethics Committee (crecscitec@sussex.ac.uk). The project reference number is ER/GM330/1. If you have any ethical concerns, please contact the ethics chair or project supervisor. University of Sussex has insurance in place to cover its legal liabilities in respect of this study.

Participant ID____________

CONSENT (COPY FOR THE RESEARCHER)

- I understand that by giving verbal or written consent below I am agreeing to take part in this University of Sussex research, and that I have read and understood this information sheet.
- I understand that my participation is entirely voluntary, that I can choose not to participate in part or all of the study, and that I can withdraw at any stage of testing without having to give a reason and without being penalised in any way.
- I understand I can request without penalty that my data be withdrawn and deleted even after testing is complete, any time up until the results are analysed (01/10/2017). I will contact you at noise.survey@sussex.ac.uk if I desire to do so, quoting the questionnaire ID at the top of the page.
- I consent to the processing of my personal information provided (age, gender etc) for the purposes of this research study. I understand that such information will be treated as strictly confidential (subject to legal limitations) and handled in accordance with the Data Protection Act 1998.
- I understand that my collected data will be stored in a de-identified way (e.g. using ID numbers not names), and kept separate from other details about me (e.g. from the consent form). Electronic data will be stored on a password-protected computer, and hard-copies will be stored behind a locked door. De-identified data may be made publicly available through ‘Sussex Research Online’ online data repository alongside publications.
- I understand that my identity will remain confidential in any written reports of this research, and that no information I disclose will lead to the identification in those reports of any individual either by the researchers or by any other party, without first obtaining my written permission.
- I understand that my name and data will not be shared with any third party outside the research group, unless I later provide written permission.

❑ Tick here if you are providing a recorded consent

Read **“My name is ____________, my participant ID is _________________ and I consent to be part of this study.”**

❑ Tick here if you are providing a written consent

________________________   ________________   _____________________

Name of Participant   Date   Signature

Date ___/___/2017 Interviewer___________ Participant ID___________

Introduction Hello my name is ______ and I am from the University of Sussex. We're doing a study in the local community on the perception of plane height, could we ask you some questions about the next few planes we see in the sky, and your general opinion about plane noise here? It would take about 15 minutes. Please hold this chart, as some of the questions refer to it.

Location__________________ **Start time** ___________

Weather conditions ❑ Clear ❑ <50% cover ❑ >50% cover
❑ Cloudy Avg. Cloud height ______________

1) Are you local to the area? ❑ Yes ❑ No

2) Did you hear that we were conducting this study?
❑ Yes ❑ No

❑ Our letter in the post	❑ A neighbour/friend
❑ Local authority/parish	❑ Website
❑ Media/Social network	❑ Other

'FLYING OVER' YOU --

3) Looking on the chart, what angles would you consider a plane to be **still flying over** the person?
❑ **10** ❑ **20** ❑ **30** ❑ **40** ❑ **50** ❑ **60**

4) Overall, what proportion of flights that you notice would you consider **flying over** you?

❑ None	❑ Some
❑ Half	❑ Most
❑ All of them	❑ Do not know

THINKING ABOUT TODAY--

5) Are there normally this **amount** of planes, or is it more or less busy than usual?

❑ Much less	❑ Less
❑ Average	❑ More
❑ Much more	

6) Are the planes normally flying at this **height**, or are they higher or lower than usual?

❑ Much lower	❑ Lower
❑ Average	❑ Higher
❑ Much higher	

7) Are the planes normally at this **volume**, or are they louder or quieter than usual?

❑ Far quieter	❑ Quieter
❑ Normal	❑ Louder
❑ Far louder	

Time : ____________
Casper ID: ____________
HEIGHT: ____________

A) How large is it on the chart when it's closest to us?
❑ **A** ❑ **B** ❑ **C** ❑ **D** ❑ **E** ❑ **F**
❑ **G** ❑ **H** ❑ **I** ❑ **J** ❑ **K**

B) These are some of the planes you might see here, which of those would you say it was?
❑ **1** ❑ **2** ❑ **3** ❑ **4**
❑ **5** ❑ **6** ❑ **7**

C) If clouds today are at _____ft, how high was that plane in feet?

❑ 500	❑ 1,000	❑ 1,500
❑ 2,000	❑ 2,500	❑ 3,000
❑ 3,500	❑ 4,000	❑ 4,500
❑ 5,000	❑ 5,500	❑ 6,000

D) Out of 5, how annoying would you say that **plane's noise** was?
❑ **0** ❑ **1** ❑ **2** ❑ **3** ❑ **4** ❑ **5**

E) Was this plane **more or less** annoying than the average?

❑ Much less	❑ Less
❑ Average	❑ More
❑ Much more	

F) Would you say this plane was any **lower** or **higher** than the average? *(elaborate if needed)*

❑ Much lower	❑ Lower
❑ Average	❑ Higher
❑ Much higher	

G) Would you say the physical size of that plane is any **smaller** or **larger** than the average?

❑ Much smaller	❑ Smaller
❑ Average	❑ Larger
❑ Much larger	

H) Was it **"flying over us"?**
❑ Yes ❑ No

OUTLIERS ---

8) Was there any specific period when you particularly noticed planes in the past 24 hours?

❑ **No** ❑ **Yes** (answer all '8.xx' questions)

8.1) When was that? Time: __________________

8.2) Does it happen every day? ❑ Yes ❑ No

8.3) Where approximately?

❑ Home, inside	❑ Home, outside
❑ Work, inside	❑ Work, outside
❑ Leisure, inside	❑ Leisure, outside
❑ Prefers not to say	

8.4) Did it disturb you from doing anything?

❑ Resting/sleeping	❑ Working/studying
❑ Gardening/sport	❑ Leisure/activity with friends
❑ Prefer not to say	❑ Other:

8.5) Out of 5, how annoying would you say that one was?

❑ **0** ❑ **1** ❑ **2** ❑ **3** ❑ **4** ❑ **5**

8.6) What was the most noticeable aspect about it?

❑ Loudness	❑ Height
❑ Size	❑ Time
❑ Frequency of flights	❑ Sound quality
❑ Tonal component	❑ Other:

9) What time of the day do you tend to be most annoyed by planes?

Time: __________________ ❑ Tick if '**not annoyed**'

10) How many *extremely* low or noisy planes do you tend to notice in a day?

Amount: ________________

11) Over the last 5 years, have planes generally become **lower** or **higher** at all?

❑ Much lower	❑ Lower
❑ Same	❑ Higher
❑ Much higher	

Time : ____________
Casper ID: ____________
HEIGHT: ____________

A) How large is it on the chart when it's closest to us?

❑ **A** ❑ **B** ❑ **C** ❑ **D** ❑ **E** ❑ **F**
❑ **G** ❑ **H** ❑ **I** ❑ **J** ❑ **K**

B) These are some of the planes you might see here, which of those would you say it was?

❑ **1** ❑ **2** ❑ **3** ❑ **4**
❑ **5** ❑ **6** ❑ **7**

C) If clouds today are at _____ft, how high was that plane in feet?

❑ 500	❑ 1,000	❑ 1,500
❑ 2,000	❑ 2,500	❑ 3,000
❑ 3,500	❑ 4,000	❑ 4,500
❑ 5,000	❑ 5,500	❑ 6,000

D) Out of 5, how annoying would you say that **plane's noise** was?

❑ **0** ❑ **1** ❑ **2** ❑ **3** ❑ **4** ❑ **5**

E) Was this plane **more or less** annoying than the average?

❑ Much less	❑ Less
❑ Average	❑ More
❑ Much more	

F) Would you say this plane was any **lower** or **higher** than the average? *(elaborate if needed)*

❑ Much lower	❑ Lower
❑ Average	❑ Higher
❑ Much higher	

G) Would you say the physical size of that plane is any **smaller** or **larger** than the average?

❑ Much smaller	❑ Smaller
❑ Average	❑ Larger
❑ Much larger	

H) Was it **"flying over us"?**

❑ Yes ❑ No

Date ___/___/2017 Interviewer____________ Participant ID____________

YOURSELF --

12) Gender: ❑ **Male** ❑ **Female** ❑ **Notes:** ___________

13) Age range**:**

❑ Under 25	❑ 25-35	❑ 36-45
❑ 46-55	❑ 56-65	❑ Over 65

14) What is your **employment** status?

❑ Employed: full time	❑ Working from home
❑ Employed: part-time	❑ Retired
❑ Self-employed	❑ Carer/family/pregnant
❑ Student: full time	❑ Other
❑ Student: part-time	❑ Prefer not to say

15) Would you say you were **sensitive to noise?**

❑ Not at all	❑ Slightly	❑ Moderately
❑ Very	❑ Extremely	❑ Don't know

AT HOME ---

16) When you are indoors at home, how much **control** do you feel you have over the impact of outside noise? (e.g. shutting windows, using headphones, or visiting quieter rooms)

❑ Not at all	❑ Slight	❑ Moderate
❑ A lot	❑ Complete control	❑ Don't know

17) When you are indoors at home, how much does **noise from planes, bother, disturb,** or **annoy you**?

❑ Not at all	❑ Slightly	❑ Moderately
❑ Very	❑ Extremely	❑ Don't know
❑ Don't hear it		

18) When you are indoors at home, how much does **noise from planes disturb your sleep?**

❑ Not at all	❑ Slightly	❑ Moderately
❑ A lot	❑ All the time	❑ Don't know
❑ Don't hear it		

19) What **type of home** do you currently live in?

❑ House: detached	❑ Bungalow: detached
❑ House: semi-detached	❑ Bungalow: semi-detached
❑ House: mid-terrace	❑ Bungalow: mid-terrace
❑ Flat	❑ Maisonette (2+ floors)
❑ Other:	

20) Is your home **insulated** against noise? ❑ Yes ❑ No

Time : ____________
Casper ID: ____________
HEIGHT: ____________

A) How large is it on the chart when it's closest to us?
❑ **A** ❑ **B** ❑ **C** ❑ **D** ❑ **E** ❑ **F**
❑ **G** ❑ **H** ❑ **I** ❑ **J** ❑ **K**

B) These are some of the planes you might see here, which of those would you say it was?
❑ **1** ❑ **2** ❑ **3** ❑ **4**
❑ **5** ❑ **6** ❑ **7**

C) If clouds today are at _____ft, how high was that plane in feet?

❑ 500	❑ 1,000	❑ 1,500
❑ 2,000	❑ 2,500	❑ 3,000
❑ 3,500	❑ 4,000	❑ 4,500
❑ 5,000	❑ 5,500	❑ 6,000

D) Out of 5, how annoying would you say that **plane's noise** was?
❑ **0** ❑ **1** ❑ **2** ❑ **3** ❑ **4** ❑ **5**

E) Was this plane **more or less** annoying than the average?

❑ Much less	❑ Less
❑ Average	❑ More
❑ Much more	

F) Would you say this plane was any **lower** or **higher** than the average? *(elaborate if needed)*

❑ Much lower	❑ Lower
❑ Average	❑ Higher
❑ Much higher	

G) Would you say the physical size of that plane is any **smaller** or **larger** than the average?

❑ Much smaller	❑ Smaller
❑ Average	❑ Larger
❑ Much larger	

H) Was it **"flying over us"?**
❑ Yes ❑ No
