# Supplementary material for: Soundscape Assessment of Aircraft Height and Size
Source: Front Psychol. 2018 Dec 18;9:2492. doi: 10.3389/fpsyg.2018.02492 (PMC6305372; doi:10.3389/fpsyg.2018.02492)

VISUAL PLANE CHART

To be held at normal reading distance (45cm)

TO BE USED DURING FIELD INTERVIEWS

1) Visual size

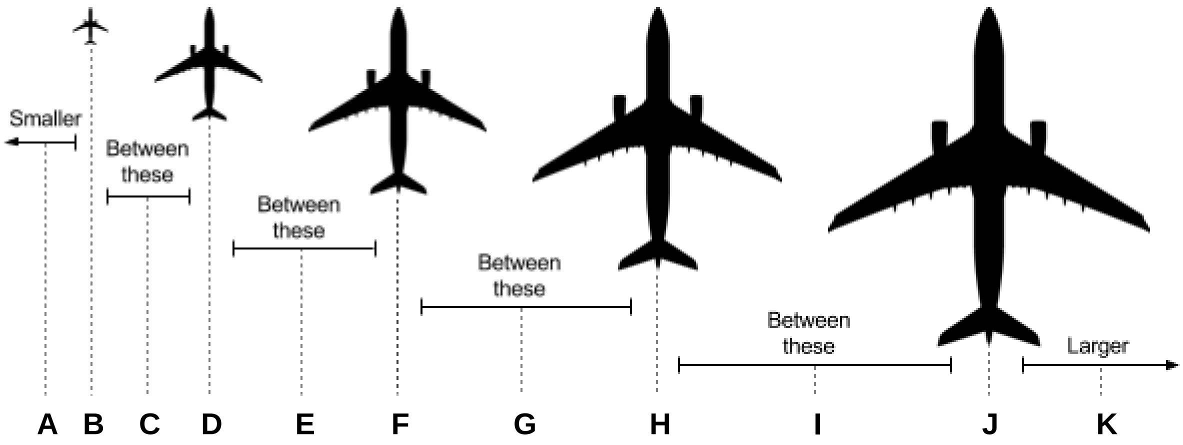

2) Which plane? (All planes are in scale)

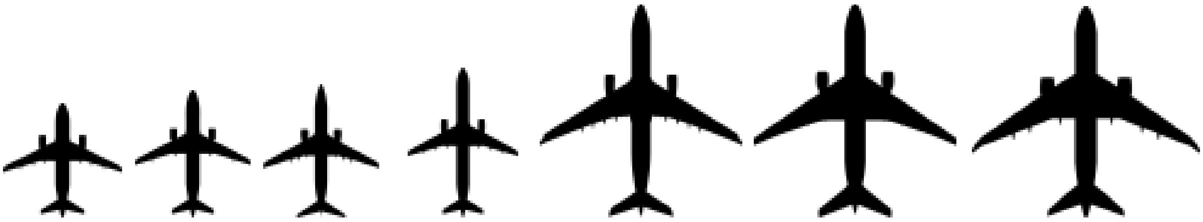

1	2	3	4	5	6	7

3) References for height measurement

For Reference (not to scale)

Heights:

Fluffy white clouds	**1,200 - 6,500 ft**
The London Shard	**1,000 ft**
Towerblock (33 floors)	**500 ft**
Plane length	**100-150 ft**
House (2 floors)	**25 ft**

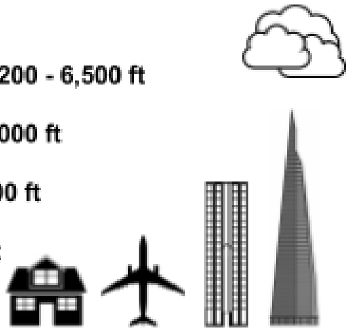

4) At what degree does the plane stop ‘flying over’ the person?

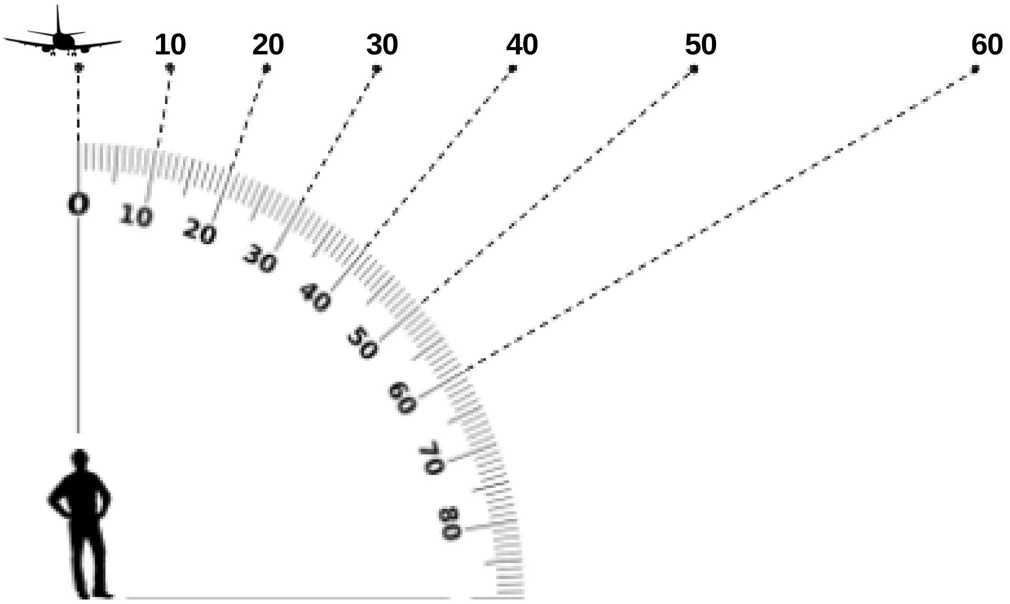

Supplement: Supplementary file 3 [file Data_Sheet_3.PDF]
