# Supplementary material for: Soundscape Assessment of Aircraft Height and Size
Source: Front Psychol. 2018 Dec 18;9:2492. doi: 10.3389/fpsyg.2018.02492 (PMC6305372; doi:10.3389/fpsyg.2018.02492)
Supplement: Supplementary file 4 [file Data_Sheet_4.pdf]

Supplementary Material

Soundscape assessment of aircraft height and size

Gianluca Memoli*, Giles Hamilton-Fletcher, Steve Mitchell

*** Correspondence:** Corresponding Author: g.memoli@sussex.ac.uk

1 Detailed description of the postal questionnaire

The postal questionnaire included the assessment of responses to transportation and environmental sounds, such as annoyance and disturbances, variables of quality of life, potential modulating variables and co-determinants, variables concerning residential conditions (e.g. sound insulation, type of house) and demographics. Here are some additional considerations on its design (see supplementary Data Sheet 1 for the postal questionnaire):

a. Self-reported sensitivity to acoustic stimuli is believed to be the major parameter affecting the perception of plane sounds (Civil Aviation Authority, 2017), followed by perceived control, gender and employment status (European Network of Noise and Health, 2013). We decided to assess sensitivity using the Weinstein scale (WNS-6B) (Kishikawa, et al., 2006). We preferred this method to the single question ("Would you say you were sensitive to noise?") used in other studies (Schreckenberg et al., 2016) (Civil Aviation Authority, 2017) for two reasons: 1) it adds to this single question different parameters, like "being easily awakened by noise"; 2) it has been shown to be more effective when an interviewer is not present (Hiroe, et al., 2017).

b. The postal questionnaire starts with an introductory set of questions, which investigates whether the respondent has participated to an acoustic survey before or has filed a complaint. This section was conceived mainly an "ice-breaker" (i.e., to focus the responder's mind on the task ahead) and helped us putting the respondent in context. At the end of this section, we ask the respondent to write down the date when the questionnaire was filled.

c. The following section A, titled "Yourself", asks about age, gender and employment status.

d. Section B, titled "Your circumstances", investigates how long the respondent had lived at the present address/village, the type of home he/she lived in and how much he/she liked living there. This was useful not only to establish expectations on the local acoustic environment, but also because some of the later questions related to perception changes in the last 5 years[1].

[1] As discussed in the Gatwick Arrivals Review (Redeborn & Lake, 2016), reports of arriving aircraft flying at lower heights started in 2013, when Gatwick increased the distance from which approaching planes could be directed by Air Traffic control (ATC), thus increasing the traffic overflying some areas and increasing the related annoyance. The choice of a comparing with 1 year and 5 years prior is therefore related to a local factor to Gatwick: it is a way of measuring the "decay

e. Section B ends with a set of questions to assess the (perceived) agency towards unwanted sounds outside i.e. the feeling of "being in control" when indoors. We start asking whether the respondent has any form of noise insulation at home (question B5), then in which period of the day he/she is mostly "aware of noise outside" (B7) and finish questioning "When indoors, how much *control* do you have on the impact of noise?" (B8). As mentioned above, agency (or "perceived control") is believed to be one of the main parameters influencing annoyance, but we found no evidence that a multi-statement test (Schreckenberg et al., 2016) could be better than a single question.

f. Section C contains a set of questions specific to this study on perception of height and size for the "average" and the "lowest" plane (see next subsection). The section ends with the question "Is plane noise less or more noticeable than all other noises (traffic, neighbors, animals etc.)?" (question C11) that prepares the participant to next section, on annoyance due to diverse sources.

g. Section D, titled "Noise over the past 12 months", reports different variations of the classical annoyance question "When indoors, how much has noise from source X bothered, disturbed or annoyed you?" (questions D2-D5). We decided to start the section asking how much the respondent likes the sounds coming from outside when indoors at home (question D1). We chose to position this question at the start of section D, to clearly differentiate positive sounds from unwanted ones and therefore preserve a neutral judgement on what the respondent may call "noise" (Payne, et al., 2009).

2 Investigating the distance-size relationship

Supplementary Figure 1 shows the ratio between the perceived size of a plane and its true size (as determined, in post-processing, by radar tracking data).

The graph shows that the error tends to increase with visual distance, but there is not a clear correlation visible to assess whether this effect is like the moon illusion (Hershenson 1989).

time" of a sudden change in annoyance, as those resulting from major change in aircraft movements (Janssen & Hong, 2017). Other airports will need a different temporal reference.

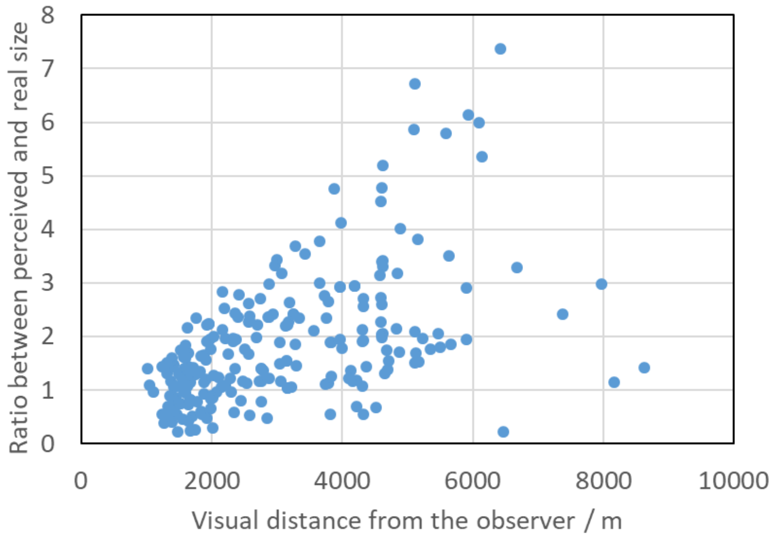

Supplementary Figure 1. Ratio between the perceived size of a plane and its true size (as determined, in post-processing, by radar tracking data), as a function of the plane-observer distance. Data relative to 242 planes over 242.

References

Civil Aviation Authority. 2017. "CAP 1506: Survey of Noise Attitudes 2014: Aircraft." Accessed October 2017. https://publicapps.caa.co.uk/docs/33/CAP%201506%20FEB17.pdf.

European Network of Noise and Health. 2013. "ENNAH's final report." Accessed October 2017. http://www.ennah.eu/final-report?lang=en.

Hershenson, Maurice. 1989. "That most puzzling illusion." In *The Moon Illusion*, edited by Maurice Hershenson. Taylor and Francis.

Hiroe, Masaaki, Koichi Makino, Saburo Ogata, and Shôsuke Suzuki. 2017. "A questionnaire survey on health effects of aircraft noise for residents living in the vicinity of Narita International Airport: the results of physical and mental health effects." *12th ICBEN Congress on Noise as a Public Health Problem.* Zurich, Switzerland.

Kishikawa, H., T. Matsui, I. Uchiyama, M. Miyakawa, K. Hiramatsu, and S.A. Stansfeld. 2006. "The development of Weinstein's noise sensitivity scale." *Noise and Health* 8 (33): 154-160.

Payne, S.R., W. Davies, and M. Adams. 2009. "DEFRA: Research into the Practical and Policy Applications of Soundscape Concepts and Techniques in Urban Areas (NANR 200)." October. Accessed October 2017. randd.defra.gov.uk/Document.aspx?Document=NO0217_8424_FRP.pdf.

Schreckenberg et al. 2016. "Effects of aircraft noise on annoyance and sleep disturbances before and after expansion Frankfurt airport - results of the NORAH study, WP1 'Annoyance and quality of life'." *INTERNOISE.* Hamburg, Germany.
